# Chloro- and Dichloro-methylsulfonyl Nitrenes: Spectroscopic Characterization, Photoisomerization, and Thermal Decomposition

**DOI:** 10.3390/molecules23123312

**Published:** 2018-12-13

**Authors:** Yang Yang, Xianxu Chu, Yan Lu, Manabu Abe, Xiaoqing Zeng

**Affiliations:** 1 College of Chemistry, Chemical Engineering and Materials Science, Soochow University, Suzhou 215123, China; 20174209103@stu.suda.edu.cn (Y.Y.); 20164209104@stu.suda.edu.cn (X.C.); 20164209106@stu.suda.edu.cn (Y.L.); 2 Department of Chemistry, Graduate School of Science, Hiroshima University, 1-3-1 Kagamiyama, Higashi-Hiroshima, Hiroshima 739-8526, Japan

**Keywords:** azides, nitrenes, decomposition, matrix isolation, photoisomerization, reaction mechanism

## Abstract

Chloro- and dichloro-methylsulfonyl nitrenes, CH_2_ClS(O)_2_N and CHCl_2_S(O)_2_N, have been generated from UV laser photolysis (193 and 266 nm) of the corresponding sulfonyl azides CH_2_ClS(O)_2_N_3_ and CHCl_2_S(O)_2_N_3_, respectively. Both nitrenes have been characterized with matrix-isolation IR and EPR spectroscopy in solid N_2_ (10 K) and glassy toluene (5 K) matrices. Triplet ground-state multiplicity of CH_2_ClS(O)_2_N (|*D*/*hc*| = 1.57 cm^−1^ and |*E*/*hc*| = 0.0026 cm^−1^) and CHCl_2_S(O)_2_N (|*D*/*hc*| = 1.56 cm^−1^ and |*E*/*hc*| = 0.0042 cm^−1^) has been confirmed. In addition, dichloromethylnitrene CHCl_2_N (|*D*/*hc*| = 1.57 cm^−1^ and |*E*/*hc*| = 0 cm^−1^), formed from SO_2_-elimination in CHCl_2_S(O)_2_N, has also been identified for the first time. Upon UV light irradiation (365 nm), the two sulfonyl nitrenes R–S(O)_2_N (R = CH_2_Cl and CHCl_2_) undergo concomitant 1,2-R shift to *N*-sulfonlyamines R–NSO_2_ and 1,2-oxygen shift to *S*-nitroso compounds R–S(O)NO, respectively. The identification of these new species with IR spectroscopy is supported by ^15^N labeling experiments and quantum chemical calculations at the B3LYP/6-311++G(3df,3pd) level. In contrast, the thermally-generated sulfonyl nitrenes CH_2_ClS(O)_2_N (600 K) and CHCl_2_S(O)_2_N (700 K) dissociate completely in the gas phase, and in both cases, HCN, SO_2_, HCl, HNSO, and CO form. Additionally, ClCN, OCCl_2_, HNSO_2_, •NSO_2_, and the atmospherically relevant radical •CHCl_2_ are also identified among the fragmentation products of CHCl_2_S(O)_2_N. The underlying mechanisms for the rearrangement and decomposition of CH_2_ClS(O)_2_N and CHCl_2_S(O)_2_N are discussed based on the experimentally-observed products and the calculated potential energy profile.

## 1. Introduction

Nitrenes R–N are neutral species containing monovalent nitrogen atoms [1]. Chemically, nitrenes are highly reactive intermediates that have been extensively used in chemical transformations such as the well-known aziridination and C–H amidation reactions, and also in the covalent functionalization of nanomaterials [2,3,4]. Typically, nitrenes R–N can be readily generated from the decomposition of azides R–N_3_ upon either photolysis or pyrolysis, in which molecular nitrogen is the only byproduct [5]. However, due to inherent instability and also high reactivity of nitrenes, the associated rapid intramolecular rearrangement and/or intermolecular reactions with solvent molecules in solution render their direct observations challenging. Therefore, conventional chemical trapping reactions have been frequently used to probe the structure and reactivity of nitrenes [6,7]. The direct characterization of nitrenes requires either ultrafast [8,9,10] or cryogenic matrix-isolation spectroscopic methods [11,12,13]. Recently, relatively more stable nitrenes stabilized by bulky organic ligands have also been synthesized as isolable compounds even at room temperatures [14].

Compared to the intensively studied alkyl [15], aryl [16,17], and carbonyl nitrenes [18,19,20], knowledge about the fundamental properties of sulfonyl nitrenes RS(O)_2_–N is limited, although this class of nitrenes have been frequently proposed as the key intermediate in the synthesis of *N*-sulfonyl-1,2,3-triazoles [21], biologically relevant sulfonamides [22], and diamines [23], where sulfonyl azides RS(O)_2_–N_3_ were usually used as the reagents. As the most frequently studied targets, naphthyl [8,10], phenyl [9], and methyl-substituted [9] sulfonyl nitrenes have already been directly observed in the photolytic decomposition of the corresponding azide precursors in solution by using ultrafast IR spectroscopy, and triplet ground-state multiplicity has been also established with EPR spectroscopy. Interestingly, experimental studies by chemical trapping [24] and ultrafast kinetics [8,9,10] in solutions have revealed that sulfonyl nitrenes are generally more rigid than carbonyl nitrenes RC(O)–N, since the latter may undergo facile rearrangement to isocyanate RNCO through 1,2-R shift [18]. In contrast, similar 1,2-R shift in sulfonyl nitrenes to *N*-sulfonlyamines R–NSO_2_, known as pseudo-Curtius rearrangement, has been only occasionally observed in the photochemistry of sulfonyl azides under matrix-isolation conditions [11,25,26]. Moreover, *S*-nitroso compounds R–S(O)NO, formally regarded as the 1,2-oxygen shifted isomers of sulfonyl nitrenes R–S(O)_2_N, have been identified among the photolysis products of fluorinated sulfonyl nitrenes such as CF_3_S(O)_2_–N [12] and FS(O)_2_–N [13].

In addition to the complex photochemistry of sulfonyl nitrenes in solution and cryogenic matrices, their thermal decompositions were found to yield diverse fragments, thus providing access to some highly reactive species in the gas phase. For instance, the thermolysis of FS(O)_2_–N [11] yields sulfonyl radical FSO_2_• and N_2_ via nitrene dimerization. Fragmentation of CH_3_S(O)_2_–N and CF_3_S(O)_2_–N [27] furnishes iminyl radical •NSO_2_ through homolytic C–S bond cleavage. Flash vacuum pyrolysis of CH_3_OS(O)_2_–N [26] and PhS(O)_2_–N [28] forms HNSO_2_ and Ph–N with concerted elimination of CH_2_O and SO_2_, respectively.

The distinct photolytic and thermal chemistry of various sulfonyl nitrenes prompted us to extend our studies to other sulfonyl nitrenes, including the barely investigated chlorinated methylsulfonyl nitrenes CH_n_X_3-n_ClS(O)_2_N (*n* = 0–2), the key intermediates in the decomposition of the synthetically useful chloromethylsulfonyl azides [29]. Herein, we report the first generation and spectroscopic characterization of triplet sulfonyl nitrenes CH_2_ClS(O)_2_N and CHCl_2_S(O)_2_N in cryogenic matrices. In addition to the photolytic rearrangement products *N*-sulfonlyamines R–NSO_2_ and *S*-nitroso compounds R–S(O)NO, a novel triplet chloromethylnitrene species CHCl_2_N has also been identified. Furthermore, the complex decomposition of the two nitrenes in the gas phase has been presented. Unlike the dominant C–S bond cleavage in CH_3_S(O)_2_–N [27], thermal decompositions of CH_2_ClS(O)_2_N and CHCl_2_S(O)_2_N initiate mainly by the concomitant elimination of HCl and SO_2_.

## 2. Results and Discussion 

### 2.1. Photolysis of CH_2_ClS(O)_2_N_3_

**Figure 1:**
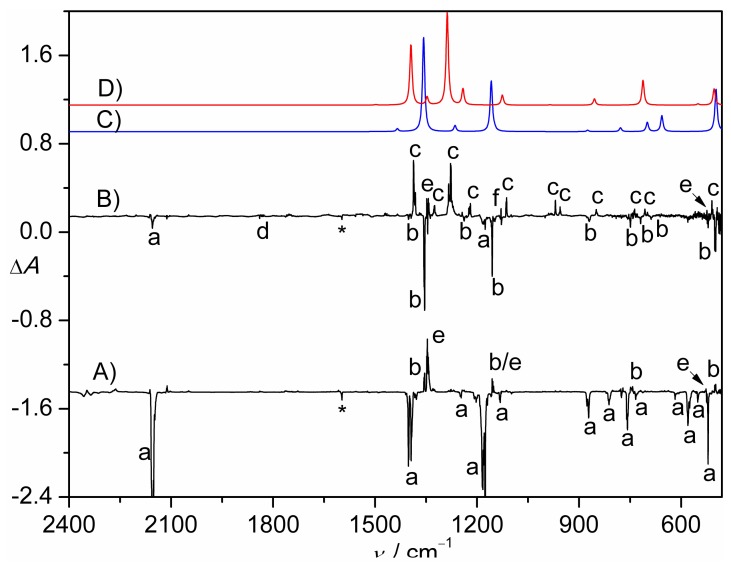
(**A**) IR difference spectrum (Absorbance, Δ*A*) showing the decomposition of CH_2_ClS(O)_2_N_3_ in N_2_-matrix upon a 193 nm laser photolysis (30 min); (**B**) IR difference spectrum (Absorbance, Δ*A*) showing the change of the N_2_-matrix upon subsequent UV-light photolysis (365 nm, 8 min). For clarity, spectrum B is 12-fold expanded along the Δ*A* axis. (**C**) Calculated IR spectrum of triplet CH_2_ClS(O)_2_N at the B3LYP/6-311++G(3df,3pd) level. (**D**) Calculated IR spectrum of CH_2_ClNSO_2_ at the B3LYP/6-311++G(3df,3pd) level. The IR bands of CH_2_ClS(O)_2_N_3_ (a), CH_2_ClS(O)_2_N (b), CH_2_ClNSO_2_ (c), CH_2_ClS(O)NO (d), SO_2_ (e), unknown species (f), and impurity H_2_O (*) are labeled. For clarity, the spectra are arbitrarily shifted along the Δ*A* axis.

The photolysis of CH_2_ClS(O)_2_N_3_ in solid N_2_-matrix was performed by using an ArF excimer laser (193 nm). The IR difference absorption spectrum reflecting the decomposition of the azide is shown in Figure 1A. Upon irradiation, nearly 42% of the azide was depleted. As a result, new species with IR bands at 1354.1, 1155.5, and 500.8 cm^−1^ formed. These band positions are close to those of SO_2_ (1347.5, 1153.1, and 524.6 cm^−1^) [30]. In addition, several weak but distinguishable IR bands in the range of 900–700 cm^−1^ appear but partially overlap with those of the azide precursor.

Given the TD-B3LYP/6-311++G(3df,3pd) calculated vertical transition at 390 nm (oscillator strength *f* = 0.0084, Table S1) for the most likely candidate species CH_2_ClS(O)_2_N, the matrix containing the 193 nm laser photolysis products of CH_2_ClS(O)_2_N_3_ was further irradiated with UV-light (365 nm). The resulting IR difference absorption spectrum (Figure 1B) suggests the depletion of trace azide but mainly the carrier for the aforementioned new IR bands (1354.1, 1155.5, and 500.8 cm^−1^). The selective depletion enables the unambiguous identification of the remaining weak IR bands for this carrier at 3023.0, 2953.6, 1398.3, 1238.6, 1128.2, 870.1, 748.8, 718.9, and 688.1 cm^−1^ (Table 1). Most of these band positions agree with the calculated IR frequencies for the expected nitrene intermediate CH_2_ClS(O)_2_N in the triplet state (Figure 1C).

According to the calculated vibrational displacement vectors of triplet CH_2_ClS(O)_2_N, the two characteristic stretching vibrations of the SO_2_ moiety are located at 1354.1 (ν_asym_(SO_2_)) and 1155.5 cm^−1^ (ν_sym_(SO_2_)), which are very close to those of other triplet sulfonyl nitrenes such as CH_3_S(O)_2_N (1349.8 and 1156.6 cm^−1^, Ne-matrix) [31], CF_3_S(O)_2_N (1387.4 and 1171.8 cm^−1^, Ar-matrix) [12], and PhS(O)_2_N (1348.9 and 1168.2 cm^−1^, Ne-matrix) [28]. No noticeable shift occurs to both IR bands in the ^15^N-labeled nitrene. Only one band at 718.9 cm^−1^ displays a large ^15^N isotopic shift of 11.1 cm^−1^, which is in good agreement with the calculated shift of 11.2 cm^−1^ for the S–N stretching mode (Table 1). It is also close to those in CH_3_S(O)_2_N (11.0 cm^−1^) [31] and CF_3_S(O)_2_N (8.2 cm^−1^) [12]. It should be noted that the calculated S–N stretching mode in singlet CH_2_ClS(O)_2_N locates at 973 cm^–1^ (Δν_cal_(^14/15^N) = 11.5 cm^−1^). Moreover, the two SO_2_ stretching vibrations in the nitrene in the singlet state at 1400 and 1053 cm^−1^ are heavily mixed with the S–N stretching mode, as evidenced by the calculated ^15^N isotopic shifts of 2.5 and 9.4 cm^−1^, respectively. The absence of these bands in the IR spectrum of the photolysis products of CH_2_ClS(O)_2_N_3_ (Figure 1) suggests that the initially-generated singlet nitrene CH_2_ClS(O)_2_N from the N_2_-elimination in the azide relaxes to the triplet ground state through rapid intersystem crossing (ISC). According to the recent ultrafast spectroscopic studies on the kinetics of arylsulfonyl nitrenes in solutions, the ISC from singlet to triplet is extremely fast (700 ± 300 ps in CCl_4_) [8,10].

As can be seen in Figure 1B, the UV-light irradiation (365 nm) results in the depletion of the nitrene CH_2_ClS(O)_2_N and another species with a weak IR band at 1839.6 cm^−1^ (labeled with d in Figure 1B). It exhibits a large ^15^N-isotopic shift of 32.1 cm^−1^. Both the band position and isotopic shift are very close to those of the most prominent N=O stretching vibration in CF_3_S(O)NO (ν(NO) = 1832.3 cm^−1^, Δν(^14/15^N) = 32.0 cm^−1^, Ar-matrix) [12]. Furthermore, they also show good agreement with the calculated strongest IR band for the oxygen-shifted rearrangement product CH_2_ClS(O)NO (ν_cal_(NO) = 1838 cm^−1^, Δν_cal_(^14/15^N) = 32.1 cm^−1^, Table S3). Nevertheless, the observation of only one band renders the identification of CH_2_ClS(O)NO tentative. In contrast, the formation of the 1,2-CH_2_Cl shifted rearrangement product CH_2_ClNSO_2_ from the 365 nm irradiation of nitrene CH_2_ClS(O)_2_N can be assured by the occurrence of one set of IR bands at 1470.3, 1387.0, 1324.7, 1278.0, 1219.7, 1113.1, 967.7, 849.4, 706.4, and 509.3 cm^−1^. Interestingly, each of these bands is accompanied with a weaker matrix-site band (Table 2), probably due to interactions of CH_2_ClNSO_2_ with the surrounding molecules in the matrix cages. The assignment is supported by the good agreement of the observed frequencies and ^15^N-isotopic shifts with the calculations (Figure 1D).

In CH_2_ClNSO_2_, the asymmetric and symmetric SO_2_ stretching vibration modes occur at 1387.0 and 1113.1 cm^−1^, respectively, and the latter strongly couples with the C–N stretching (ν(CN)), as indicated by the ^15^N-isotopic shift of 15.5 cm^−1^. The ν(N=S) stretching mode appears at 1278.0 cm^−1^ (Δν(^14^/^15^N) = 10.4 cm^−1^). It is slightly lower than that in CH_3_NSO_2_ (1294.9 cm^−1^, Ar-matrix), which has been very recently generated in the gas phase through flash vacuum pyrolysis of sulfamoyl chloride MeN(H)S(O)_2_Cl through HCl-elimination [32]. In addition to CH_2_ClNSO_2_, traces of SO_2_ were also produced. However, the counterpart fragmentation species CH_2_ClN or its isomer CHCl=NH was not observed due to weak IR intensities.

### 2.2. Flash Vacuum Pyrolysis of CH_2_ClS(O)_2_N_3_

**Figure 2:**
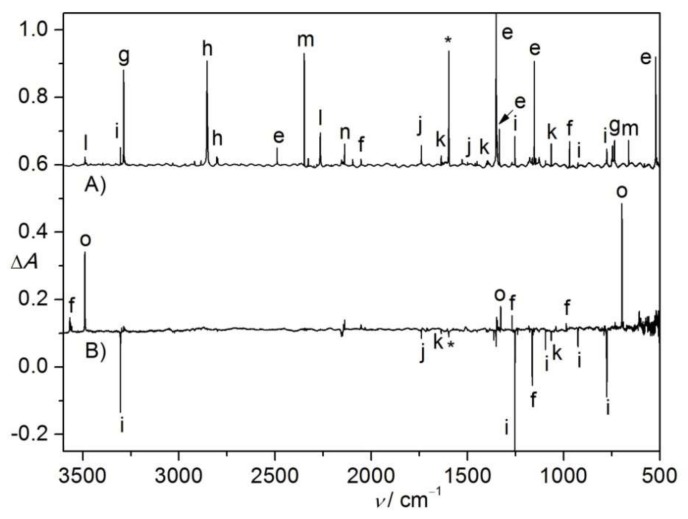
(**A**) IR spectrum (Absorbance, *A*) of the N_2_-matrix isolated flash vacuum pyrolysis (600 K) products of CH_2_ClS(O)_2_N_3_. (**B**) IR difference spectrum (Absorbance, Δ*A*) showing the change of the N_2_-matrix upon subsequent 266 nm laser photolysis (25 min). For clarity, spectrum B is 12-fold expanded along the Δ*A* axis. The IR bands of SO_2_ (e), unknown species (f), HCN (g), HCl (h), HNSO (i), H_2_CO (j), CH_2_NH (k), HNCO (l), CO_2_ (m), CO (n), HOSN (o), and impurity H_2_O (*) are labeled. For clarity, the spectra are arbitrarily shifted along the Δ*A* axis.

Similar to the photochemistry, the thermal decomposition of sulfonyl azides (e.g., FS(O)_2_N_3_ [11] and PhS(O)_2_N_3_ [28]) should also initiate by extruding molecular nitrogen, followed by secondary fragmentation of the sulfonyl nitrene intermediates. To uncover the thermal behavior of CH_2_ClS(O)_2_N, flash vacuum pyrolysis (FVP, 600 K) of CH_2_ClS(O)_2_N_3_ in N_2_ dilution (1:1000) was performed. The IR spectrum of the pyrolysis products (Figure 2A) reveals complete dissociation of the azide, fragments SO_2_ (e) [33,34], HCN (g, 3287.8 and 735.5 cm^−1^) [35], HCl (h, 2854.5, 2803.8 cm^−1^) [32], HNSO (i, 3305.0, 1253.5, 1094.9, 925.6, and 775.5 cm^−1^) [36], H_2_CO (j, 1740.0 and 1499.7 cm^−1^) [26], CH_2_NH (k, 1637.4, 1450.9, and 1064.7 cm^−1^) [35], HNCO (l, 3489.2 and 2265.7 cm^−1^) [37], CO_2_ (m, 2348.9 and 662.3 cm^−1^) [38], CO (n, 2138.9 cm^−1^) [38], and N_2_ (IR inactive) form.

The absence of the IR band for nitrene CH_2_ClS(O)_2_N implies its immediate dissociation under the FVP conditions. Unlike the straightforward C–S bond cleavage in other alkylsulfonyl nitrenes CH_3_S(O)_2_N (→•CH_3_ + •NSO_2_) and CF_3_S(O)_2_N (→•CF_3_ + •NSO_2_), the absence of the IR bands for •CH_2_Cl and •NSO_2_ in the IR spectrum (Figure 2A), and the presence of very strong IR bands for SO_2_, mean that no C–S bond cleavage, but rather, SO_2_-elimination occurs for CH_2_ClS(O)_2_N. The identification of HCl and HCN strongly suggests that further fragmentation happens to the SO_2_-elimination product CH_2_ClN. The unexpected formation of a second pair of fragments HNSO/HCl/CO from CH_2_ClS(O)_2_N can be tentatively explained by first rearrangement to CH_2_ClNSO, as followed by further HCl-elimination via the intermediacy of a putative carbene species H–C–NSO_2_. As further proof of the identification of HNSO among the products, the previously-observed [36,39] isomerization to HOSN was repeated by irradiation with a 266 nm laser (Figure 2B).

### 2.3. Photolysis of CHCl_2_S(O)_2_N_3_

**Figure 3:**
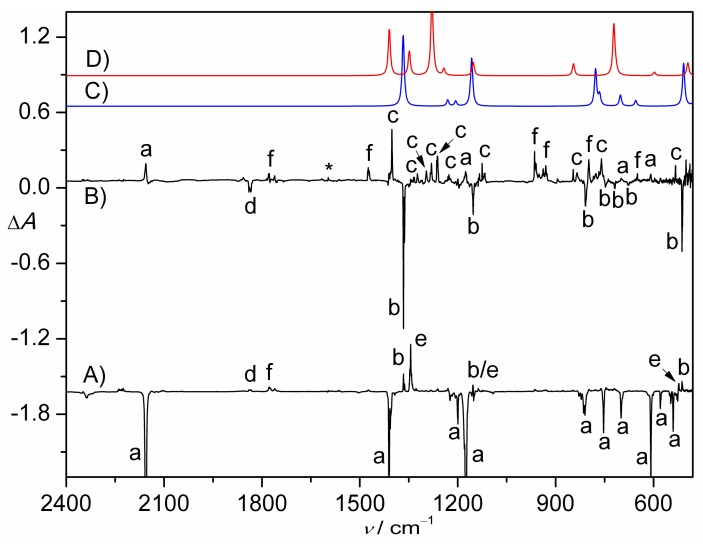
(**A**) IR difference spectrum (Absorbance, Δ*A*) showing the decomposition of CHCl_2_S(O)_2_N_3_ in N_2_-matrix upon a 193 nm laser photolysis (11 min); (**B**) IR difference spectrum (Absorbance, Δ*A*) showing the change of the N_2_-matrix upon subsequent UV-light photolysis (365 nm, 10 min). (**C**) Calculated IR spectrum of triplet CHCl_2_S(O)_2_N at the B3LYP/6-311++G(3df,3pd) level. (**D**) Calculated IR spectrum of CHCl_2_NSO_2_ (singlet-II) at the B3LYP/6-311++G(3df,3pd) level. The IR bands of CHCl_2_SO_2_N_3_ (a), CHCl_2_SO_2_N (b), CHCl_2_NSO_2_ (c), CHCl_2_S(O)NO (d), SO_2_ (e), unknown species (f), and impurity H_2_O (*) are labeled. For clarity, the spectra are arbitrarily shifted along the Δ*A* axis.

The photolysis of CHCl_2_S(O)_2_N_3_ in N_2_-matrix was also performed with a 193 nm laser. Compared to the photochemistry of CH_2_ClS(O)_2_N_3_, the depletion of CHCl_2_S(O)_2_N_3_ is more relatively efficient, since 52% of the azide vanishes in 11 min. In the corresponding IR difference absorption spectrum (Figure 3A), the IR bands of SO_2_ (1344.4, 1150.2, and 522.4 cm^−1^) and new species at 1835.1, 1777.3, 1366.5, 1153.2, and 512.6 cm^–1^ can be identified.

In order to distinguish these new IR bands, the matrix was irradiated with UV-light (365 nm), leading to the main depletion of the IR bands at 1835.1, 1366.5, 1153.2, 808.4, 747.7, 718.5, 675.8, and 512.6 cm^−1^ (Table 3). All these IR bands except the first one can be reasonably assigned to the nitrene intermediate CHCl_2_S(O)_2_N in the triplet state by comparing with the calculated IR data (Figure 3C). With the aid of the calculations, weaker bands at 3027.2, 1196.1, and 1163.7 cm^−1^ were also found to belong to the same carrier. This assignment is further supported by the observation of well-resolved ^15^N-isotopic shift of 9.6 cm^−1^ only for the S–N stretching mode (ν(SN)) at 718.5 cm^−1^. As expected, the band position is very close to that in triplet CH_2_ClS(O)_2_N at 718.9 cm^−1^. As for the IR band at 1835.1 cm^−1^, the accompanying large ^15^N-isotopic shift of 31.7 cm^−1^ suggests a tentative assignment to an NO-containing species (CHCl_2_S(O)NO, Table S5).

Upon the UV-light irradiation, CHCl_2_S(O)_2_N partially recombines molecular nitrogen and reforms CHCl_2_S(O)_2_N_3_ (Figure 3B). In the meantime, 1,2-CHCl_2_ shift occurs and furnishes CHCl_2_NSO_2_ (Figure 3D). Similar to the IR spectrum of CH_2_ClNSO_2_ (Table 2), most of the IR bands for CHCl_2_NSO_2_ (Table 4) split into doublets due to weak interactions with the surrounding molecules. Based on the distinct ^15^N-isotopic shifts, the IR bands at 1280.5/1262.7 and 1125.4/1117.0 cm^−1^ belong mainly to the ν(SN) and ν(CN) stretching modes, which are also very close to those in CHCl_2_NSO_2_ at 1283.7/1278.0 and 1114.6/1113.1 cm^−1^, respectively. Additionally, another species with IR bands at 1777.3, 1474.4, 964.5, 930.5, and 798.8 cm^−1^ appears from the UV-light photolysis of CHCl_2_S(O)_2_N, its identification remains unclear.

### 2.4. Flash Vacuum Pyrolysis of CHCl_2_S(O)_2_N_3_

**Figure 4:**
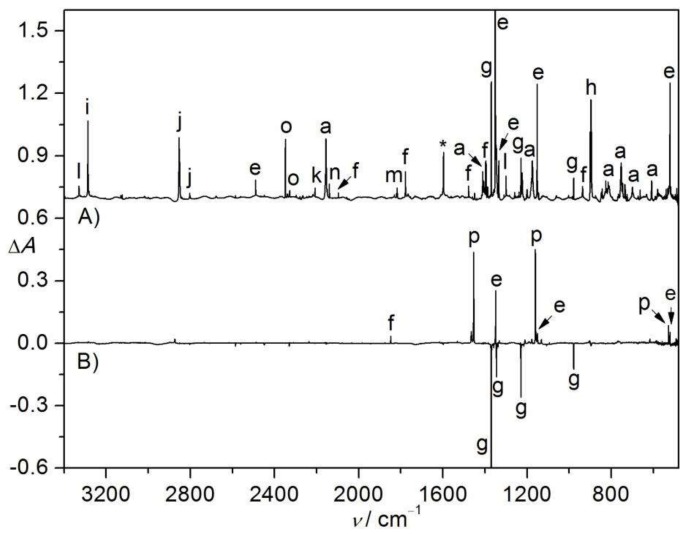
(**A**) IR spectrum (Absorbance, *A*) of the N_2_-matrix isolated flash vacuum pyrolysis (700 K) products of CHCl_2_S(O)_2_N_3_. (**B**) IR difference spectrum (Absorbance, Δ*A*) showing the change of the N_2_-matrix upon subsequent UV-light photolysis (365 nm, 10 min). The IR bands of CHCl_2_SO_2_N_3_ (a), SO_2_ (e), unknown species (f), •NSO_2_ (g), CHCl_2_ (h), HCN (i), HCl (j), ClCN (k), HNSO_2_ (l), OCCl_2_ (m), CO (n), CO_2_ (o), OSNO (p), and impurity H_2_O (*) are labeled. For clarity, the spectra are arbitrarily shifted along the Δ*A* axis.

The IR spectrum of the flash vacuum pyrolysis (700 K) products of CHCl_2_S(O)_2_N_3_ is depicted in Figure 4A. Traces of the azide survive, SO_2_, HCN, HCl, CO, HNSO, OCCl_2_ (m, 1817.9, 847.2 and 843.6 cm^−1^) [40], ClCN (k, 2208.0 cm^−1^) [41], •NSO_2_ (g, 1376.7, 1345.0, 1229.3 and 936.6 cm^−1^) [27], HNSO_2_ (l, 3329.6, 1387.9, 1300.3, and 672.1 cm^−1^) [26], and •CHCl_2_ (h, 1223.3 and 896.2 cm^−1^) [42] can be identified among the pyrolysis products. The identification of •NSO_2_ can be ascertained with the subsequent photoisomerization with OSNO• upon further UV-light irradiation (Figure 4B).

The presence of •NSO_2_ and •CHCl_2_ but no counterpart •N_3_ radicals among the FVP products of CHCl_2_S(O)_2_N_3_ clearly demonstrates that the azide decomposes by the first formation of CHCl_2_S(O)_2_N through N_2_-elimination. However, the initially generated nitrene CHCl_2_S(O)_2_N is thermally unstable, which dissociates by homolytic cleavage of the C–S bond. Alternatively, it may undergo heterolytic cleavage of the C–S bond with concerted H-migration to furnish HNSO_2_ and dichlorocarbene CCl_2_, and the latter reacts with oxygen-containing species and yields the experimentally observed OCCl_2_. Another possible pathway involves the isomerization of CHCl_2_S(O)_2_N to CHCl_2_NSO_2_, and the latter may break the C–N bond to a pair of radicals •NSO_2_/•CHCl_2_ and HNSO_2_/CCl_2_. It should be noted that the FVP of CHCl_2_S(O)_2_N_3_ provides an efficient and practical method for the gas-phase generation of the atmospherically-important radical •CHCl_2_ [43], since its production in the previous spectroscopic studies utilized either laser photolysis of CHCl_2_Br [44] or the reaction of chloroform with lithium atoms [45].

### 2.5. EPR Spectroscopy

**Figure 5:**
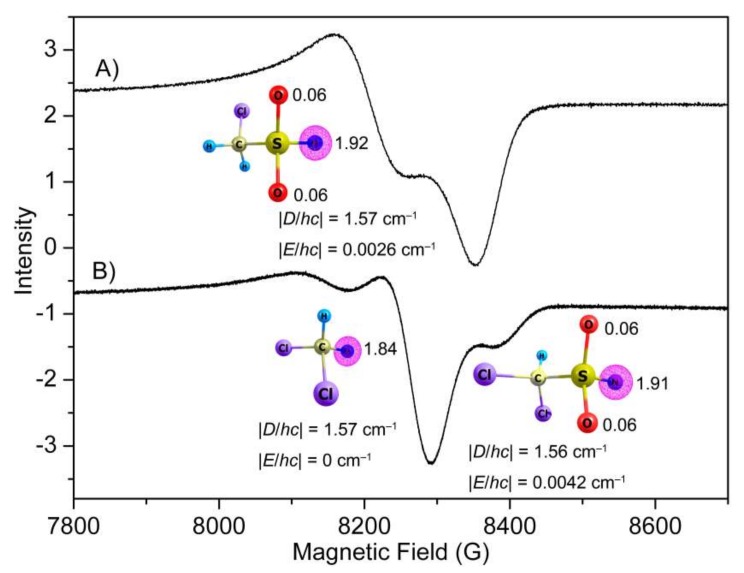
EPR spectra of the 266 nm laser photolysis products of CH_2_ClS(O)_2_N_3_ (**A**) and CHCl_2_S(O)_2_N_3_ (**B**) in glassy toluene matrices (0.4 mmol mL^−1^) at 5 K. Natural spin densities of the nitrenes computed at the M06-2X/6-311++G(3df,3pd) level are depicted.

To capture the nitrene intermediates in the decomposition of CH_2_ClS(O)_2_N_3_ and CHCl_2_S(O)_2_N_3_, the photochemistry (266 nm) of both sulfonyl azides in glassy toluene matrices (5 K) was followed with EPR spectroscopy. The obtained EPR spectra (Figure 5) demonstrate typical triplet nitrene signals in the region of 7500–9000 G at about 9.40 GHz resonance frequency. The derived zero-field splitting parameters (ZFSP) for CH_2_ClS(O)_2_N (|*D*/*hc*| = 1.57 cm^−1^ and |*E*/*hc*| = 0.0026 cm^−1^) and CHCl_2_S(O)_2_N (|*D*/*hc*| = 1.56 cm^−1^ and |*E*/*hc*| = 0.0042 cm^−1^) are very similar to those of other sulfonyl nitrenes, such as Me_2_NS(O)_2_–N (|*D*/*hc*| = 1.57 cm^−1^ and |*E*/*hc*| = 0.0038 cm^−1^) [46], CF_3_S(O)_2_–N (|*D*/*hc*| = 1.741 cm^−1^ and |*E*/*hc*| = 0 cm^−1^) [12], and FS(O)_2_N (|*D*/*hc*| = 1.620 cm^−1^ and |*E*/*hc*| = 0.0055 cm^−1^) [13]. According to the linear correlation between the calculated spin densities (ρ, CH_2_ClS(O)_2_N: 1.92; CHCl_2_S(O)_2_N: 1.91, M06-2X/6-311++G(3df,3pd)) and zero-field D values [47,48], the predicted *D* values of 1.72 and 1.70 cm^−1^ agree with the experimental observations of 1.57 and 1.56 cm^−1^, respectively. Consistent with the observed nonzero E values in CH_2_ClS(O)_2_–N and CHCl_2_S(O)_2_–N, small spin densities of about 0.12 are equally distributed on the two neighboring O atoms.

In line with the IR spectroscopic observation of SO_2_-elimination during the photolysis of CHCl_2_S(O)_2_N_3_, a second triplet nitrene signal with ZFSP of |*D*/*hc*| = 1.57 cm^−1^ and |*E*/*hc*| = 0 cm^−1^ for dichloromethylnitrene CHCl_2_N was also observed in the EPR spectrum (Figure 5B). Its assignment is supported by the close similarity with other alkyl nitrenes such as CH_3_N (|*D*/*hc*| = 1.720 cm^−1^ and |*E*/*hc*| < 0.003 cm^−1^) [49] and CF_3_N (|*D*/*hc*| = 1.741 cm^−1^ and |*E*/*hc*| = 0 cm^−1^) [12]. In contrast, no EPR signal for chloromethylnitrene CH_2_ClN could be observed in the photolysis of CH_2_ClS(O)_2_N_3_, which is probably due to immediate isomerization to singlet species (CHCl=NH) under the photolysis conditions.

### 2.6. Quantum Chemical Calculations

**Figure 6:**
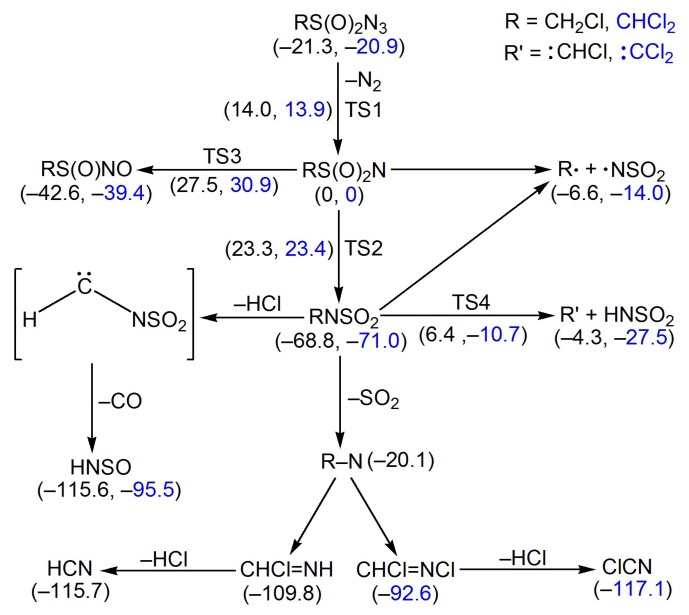
Calculated energy profile for the decomposition of RS(O)_2_N_3_ (R = CH_2_Cl and CHCl_2_) in the singlet state at the B3LYP/6-311++G(3df,3pd) level of theory. The calculated molecules structures (bond lengths in Å and angles in °) for the selected species are also depicted.

The energies for the species involving in the stepwise decomposition of CH_2_ClS(O)_2_N_3_ and CHCl_2_S(O)_2_N_3_ via the intermediacy of the corresponding nitrenes were calculated at the B3LYP/6-311++G(3df,3pd) level (Figure 6). The barriers (TS1) for the N_2_-elimination in CH_2_ClS(O)_2_N_3_ (35.3 kcal mol^−1^) and CHCl_2_S(O)_2_N_3_ (34.8 kcal mol^−1^) are comparable with those of other sulfonyl azides such as PhS(O)_2_N_3_ (35 kcal mol^−1^, CBS-QB3) [28] and CH_3_OS(O)_2_N_3_ (35 kcal mol^−1^, CCSD(T)/6-311++G(2df,2p)//UMP2/6-311++G(2df,2p)) [26], and they are also close to those for the nitrene formation in typical carbonyl azides such as FC(O)N_3_ (33 kcal mol^−1^, B3LYP/6-311+G(3df)) [50] and CH_3_OC(O)N_3_ (34 kcal mol^–1^, B3LYP/6-311++G(3df,3pd)) [51].

For the initially-generated singlet sulfonyl nitrenes RS(O)_2_N (R = CH_2_Cl and CHCl_2_), several competing processes might be responsible for their disappearance. Similar to the well-established Curtius-rearrangement of all carbonyl nitrenes (RC(O)N → RNCO) [52], RS(O)_2_N can undergo either 1,2-R shift to RNSO_2_ or 1,2-oxygen shift to RS(O)NO. Both pathways are highly exothermic, and the higher activation barriers for the latter (TS3, CH_2_ClS(O)_2_N: 27.5 kcal mol^−1^; CHCl_2_S(O)_2_N: 30.9 kcal mol^−1^) than the former (TS2, CH_2_ClS(O)_2_N: 23.3 kcal mol^−1^; CHCl_2_S(O)_2_N: 23.4 kcal mol^−1^) render their contribution in the gas phase reactions of the nitrenes unlikely. However, the excessive energy input from the ArF laser irradiation (193 nm, 148 kcal mol^−1^) [53] in the photochemistry can overcome the barrier and enable the formation of RS(O)NO as minor products. In addition to the intramolecular rearrangement, the sulfonyl nitrenes may decompose exothermically through the homolytic C–S bond cleavage to alkyl radicals R• (•CH_2_Cl and •CHCl_2_) and •NSO_2_ or heterolytic C–N bond fragmentation with concerted H-migration to carbenes HCCl and ClCCl and HNSO_2_. These two pairs of fragments may also be derived from the decomposition of RNSO_2_; however, the large C–N bond dissociation energies (CH_2_ClNSO_2_: 62.2 kcal mol^−1^; CHCl_2_NSO_2_: 57.0 kcal mol^−1^) and formidable barriers for the concerted H-migration (TS4, CH_2_ClNSO_2_: 75.2 kcal mol^–1^; CHCl_2_NSO_2_: 60.3 kcal mol^−1^) rule out these pathways under the pyrolysis conditions. In agreement with the IR detectable amounts of •CHCl_2_/•NSO_2_ and OCCl_2_/HNSO_2_ among the FVP products of CHCl_2_S(O)_2_N_3_, the energy release from the decomposition of CHCl_2_S(O)_2_N is larger than that of CHCl_2_NSO_2_. 

The absence of RNSO_2_ (R = CH_2_Cl and CHCl_2_), but the presence of HCl, CO, and HNSO, among the pyrolysis products of the azides implies further dissociation through the thermodynamically favorable HCl- or Cl_2_-elimination (Figure 6). According to the B3LYP/6-311++G(3df,3pd) calculation, the putative carbene species H–C–NSO_2_ is highly unstable in the closed-shell singlet state and prefers either N–S bond breakage to HCN and SO_2_ or further CO-elimination to HNSO. The SO_2_ formation may also be attributed to the direct N–S bond breakage in RNSO_2_. By analogy to the thermal instability of other alkyl nitrenes (e.g., CH_3_N [54] and CF_3_N [12]), the thermally-generated nitrenes CH_2_ClN and CHCl_2_N isomerize to CHCl=NH and CHCl=NCl and then eliminate HCl to yield the observed HCN and ClCN, respectively.

**Figure 7:**
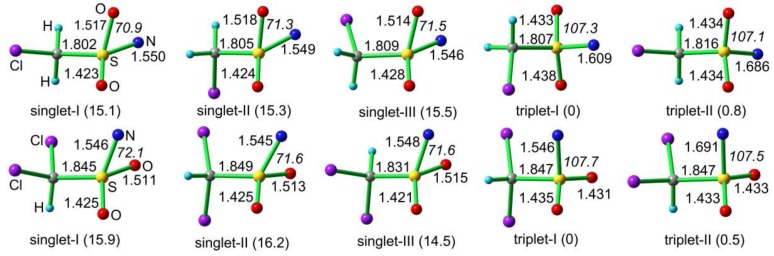
Calculated molecular structures and relative energies (in parentheses, kcal mol^−^^1^) of CH_2_ClS(O)_2_N and CHCl_2_S(O)_2_N at the B3LYP/6-311++G(3df,3pd) level. Selected bond lengths in Å and angles in ° (in italics) are given.

The calculated molecular structures and relative energies of CH_2_ClS(O)_2_N and CHCl_2_S(O)_2_N in the singlet and triplet states are depicted in Figure 7. For each nitrene in the singlet state, three rotamers differing in the staggered orientation of the methyl group with respect to the SO_2_N moiety are close-in-energy minima. Consistent with the IR and EPR spectroscopic observations, both sulfonyl nitrenes prefer triplet ground state, and the singlet state are about 15 kcal mol^−1^ higher in energy at the B3LYP/6-311++G(3df,3pd) level. The energy gaps between the singlet and triplet states (Δ*E*_ST_) for CH_2_ClS(O)_2_N (15.1 kcal mol^−1^) and CHCl_2_S(O)_2_N (14.5 kcal mol^−1^) are close to those of CH_3_S(O)_2_N (13.6 kcal mol^−1^) and PhS(O)_2_N (14.7 kcal mol^−1^) at the same theoretical level [31].

Due to intramolecular interaction between the oxygen atom with the electron-deficient nitrogen center, the singlet sulfonyl nitrenes are distorted from *C*_s_-symmetry. In the lowest-energy *C*_1_-symmetric rotamer (singlet-I) of CH_2_ClS(O)_2_N, the Cl–C and S–N bonds adopts an antiperiplanar configuration with a dihedral angle (φ(ClCSN)) of −158.8°. The φ(ClCSN) dihedral angles in singlet-II and singlet-III are 84.4 and −31.7°, respectively. Similarly, in the most stable rotamer of singlet CHCl_2_S(O)_2_N (singlet-III), the two S–N bond adopts an antiperiplanar configuration with the bisector of the ClSCl angle. In the triplet state of CH_2_ClS(O)_2_N, the more stable rotamer (triplet-I) adopts *C*_1_-symmetry with the S–N bond being synperiplanar with the Cl–C bond (φ(ClCSN) = −63.3°); however, the *C*_s_-symmetric rotamer (triplet-II) exhibits an synperiplanar conformation between these two bonds (φ(ClCSN) = 180°). Structurally, sulfonyl nitrenes in the singlet state are stabilized by the intramolecular N–O interactions, as evidenced by the considerably smaller OSN angles in the singlet state (ca. 72°) than the triplet state (ca. 107°). Unlike the much stronger intramolecular N–N interactions in singlet ground-state sulfamoyl nitrenes [46], the stabilizing N–O interactions in alkylsulfonyl nitrenes can hardly switch the spin-state. In contrast, very recent studies on structurally-related carbonyl nitrenes RC(O)N have demonstrated that the spin multiplicity can be effectively switched by the stabilizing N–O interactions [18,52].

## 3. Materials and Methods 

### 3.1. Sample Preparation

Caution! Covalent azides are explosive! Although no explosions occurred during this work, appropriate safety precautions (face shields, leather gloves, and protective leather clothing) should be taken, especially when working with pure CH_2_ClS(O)_2_N_3_ and CHCl_2_S(O)_2_N_3_ in the condensed phase.

Chloromethylsulfonyl azide, CH_2_ClS(O)_2_N_3_, was prepared by the reaction of chloromethylsulfonyl chloride with sodium azide. Briefly, acetonitrile (0.3 mL) and freshly purified chloromethylsulfonyl chloride (0.3 g, 2.0 mmol) were distilled into a glass vessel which contains dried NaN_3_ (0.2 g, 3.2 mmol). The mixture was stirred at room temperature for 18 h. The volatile crude products were separated by passing through three successive cold U-traps (−20, −80, and −196 °C). Pure azide CH_2_ClS(O)_2_N_3_ was retained in the first trap as white crystals. ^1^H-NMR (400MHz, CDCl_3_, 298 K): δ = 4.72 (s, 2H); ^13^C-NMR (400 MHz, CDCl_3_ 298 K): δ *=* 57.9 ppm; IR (gas-phase): 2148, 1412, 1246, 1183, 1135, 868, 814, 750, 577, and 524 cm^−^^1^; Raman(solid): 3032, 2961, 2161, 1391, 1185, 1163, 1131, 869, 807, 747, 736, 575, 536, 515, 451, 407, 315, and 285 cm^−^^1^. 1-^15^N sodium azide (98 atom% ^15^N, EURISO-TOP GmbH) was used for the preparation of ^15^N-labeled sample.

Dichloromethylsulfonyl azide, CHCl_2_S(O)_2_N_3_, was synthesized in a similar manner by the reaction of freshly purified dichloromethylsulfonyl chloride (0.6 g, 3.0 mmol) with NaN_3_ (0.3 g, 4.6 mmol) in acetonitrile (0.5 mL). The mixture was stirred at room temperature for 24 h. Separation of the volatile products was carried out by using three cold traps of −16, −80, and −196 °C. The desired product CHCl_2_S(O)_2_N_3_ was retained in the first trap as colorless liquid. ^1^H-NMR (400 MHz, CDCl_3_, 298 K): δ = 6.41 ppm (s, 1H); ^13^C-NMR (400 MHz, CDCl_3_, 298 K): δ *=* 79.4 ppm; IR (gas-phase): 2148, 1422, 1355, 1222, 1179, 1150, 816, 751, 694, 608, 576, and 529 cm^−^^1^; Raman (liquid): 2993, 2961, 2155, 1382, 1203, 1156, 1144, 799, 746, 698, 603, 575, 536, 452, 410, 342, 295, 238, and 211 cm^−^^1^. 1-^15^N sodium azide (98 atom % ^15^N, EURISO-TOP GmbH) was used for the preparation of ^15^N labeled sample.

### 3.2. Spectroscopy

Gas-phase IR spectra were recorded on a Bruker spectrometer (Tensor 27) (Bruker Optik GmbH, Ettlingen, Germany). Raman spectra were recorded on a Horiba JY HR800 Raman spectroscopy (HORIBA, Lille, France).

### 3.3. Matrix IR Spectroscopy

Matrix IR spectra were recordedon a FT-IR spectrometer (Bruker 70 V) in a reflectance mode using a transfer optic. A KBr beam splitter and MCT detector were used in the mid-IR region (4000−500 cm^−1^). For each spectrum, 200 scans at a resolution of 0.5 cm^−1^ were coadded. The gas sample mixed by passing a flow of N_2_ gas through a cold U-trap (CH_2_ClS(O)_2_N_3_: −5 °C; CHCl_2_S(O)_2_N_3_: −10 °C) containing ca. 10 mg of the azide. The mixture (azide/dilution gas ≈ 1:1000 estimated) was passed through an aluminum oxide (o.d. 2.0 mm, i.d. 1.0 mm), which could be heated over a length of approximately 25 mm by a tantalum wire (o.d. 0.4 mm, resistance 0.4 Ω). Then, the mixture was immediately deposited (2 mmol/h) onto the Rh-plated copper block matrix support (15 K) in a high vacuum (~10^−6^ Pa). While not directly measured, the expected residence time of the mixture in the pyrolysis tube is about a few milliseconds, and the pressure inside the pyrolysis tube is about 10 mbar. The electric power (voltage/current) used in pyrolysis experiments was 4.0 V/1.9 A for CH_2_ClS(O)_2_N_3_ and 4.5 V/2.9 A for CHCl_2_S(O)_2_N_3_. Photolysis experiments were performed with ArF excimer laser (GAM LASER, Orlando, FL, USA) (193 nm, Gamlaser EX5/250, 5 mJ, 3 Hz), Nd^3+^: YAG laser (266 nm, MPL-F-266, 10 mW), and UV lamp (365 nm).

### 3.4. Computational Details

Geometry optimizations were performed using DFT-B3LYP [55] method combined with the 6-311++G(3df,3pd) basis set. Time-dependent (TD) DFT (B3LYP/6-311++G(3df,3pd)) [56,57] calculations were performed for the prediction of UV−vis transitions. Local minima were confirmed by vibrational frequency analysis, and transition states were further confirmed by intrinsic reaction coordinate (IRC) calculations [58,59]. All the calculations were performed using the Gaussian 09 software package (Gaussian, Inc., Wallingford, CT, USA) [60].

## 4. Conclusions

The UV laser photolytic (193 and 266 nm) and thermal decomposition of two alkylsulfonyl azides RS(O)_2_N_3_ (R = CH_2_Cl and CHCl_2_) have been studied by combining matrix-isolation IR and EPR spectroscopy and quantum chemical calculations. Two new sulfonyl nitrenes CH_2_ClS(O)_2_N and CHCl_2_S(O)_2_N in the triplet ground state have been directly observed and spectroscopically characterized. Upon subsequent UV light irradiations, both nitrenes undergo rearrangement reactions to the corresponding *N*-sulfonlyamines R–NSO_2_ and 1,2-oxygen shift to *S*-nitroso compounds R–S(O)NO in solid N_2_-matrices. In the gas phase, the monochloro-substituted sulfonyl nitrene CH_2_ClS(O)_2_N prefers rearrangement to CH_2_ClNSO_2_ with subsequent decomposition to HCl, HNSO, and CO, in which an intriguing carbene species H–C–NSO_2_ might be involved. The dichloro-substituted sulfonyl nitrene CHCl_2_S(O)_2_N partially undergoes homolytic C–S bond cleavage to a pair of radicals •CHCl_2_ and •NSO_2_. Its rearrangement product CHCl_2_NSO_2_ also decomposes to HNSO_2_/CCl_2_ and HCl/SO_2_/ClCN.

## Figures and Tables

**Table 1:** Calculated and observed IR frequencies (>500 cm^−1^) of CH_2_ClS(O)_2_N.

Calculated ^a^				Observed ^b^		Assignment ^c^
Singlet		Triplet		N_2_-matrix		
ν	Δν	ν	Δν	ν	Δν	
3183 (4)	0.0	3176 (2)	0.0	3023.0 (4)	<0.5	ν_asym_(CH_2_)
3098 (5)	0.0	3097 (3)	0.0	2953.6 (7)	<0.5	ν_sym_(CH_2_)
1431 (5)	0.0	1435 (4)	0.0	1398.3 (3)	<0.5	δ(CH_2_)
1400 (164)	2.5	1357 (155)	0.0	1354.1 (100)	<0.5	ν_asym_(SO_2_)
1268 (13)	0.1	1265 (10)	0.0	1238.6 (6)	<0.5	*ω*(CH_2_)
1161 (1)	0.0	1158 (83)	0.0	1155.5 (40)	<0.5	ν_sym_(SO_2_)
1053 (67)	9.4	1149 (2)	0.0	1128.2 (5)	<0.5	τ(CH_2_)
973 (12)	11.5	875 (2)	0.0	870.1 (8)	<0.5	ρ(CH_2_)
870 (<1)	0.1	778 (6)	0.3	748.8 (9)	<0.5	ν(CCl)
761 (21)	0.5	699 (15)	11.2	718.9 (6)	11.1	ν(SN)
686 (16)	0.9	656 (26)	0.2	688.1 (7)	<0.5	ν(SC)
515 (83)	3.0	497 (69)	4.0	500.8 (42)	<0.5	δ(SO_2_)

^a^ The calculated IR harmonic frequencies (ν, unscaled), ^15^N-isotopic shifts (Δν), and band intensities (km mol^−1^, in parentheses) for the singlet and triplet states at the B3LYP/6-311++G(3df,3pd) level. Full list of the calculated IR frequencies for all the conformers are given in Table S2. ^b^ The observed band positions (ν), ^15^N-isotopic shifts (Δν), and relative intensities (in parentheses) in N_2_-matrix. ^c^ Tentative assignment based on the calculated vibrational displacement vectors for the triplet state.

**Table 2:** Calculated and observed IR frequencies (>500 cm^–1^) of CH_2_ClNSO_2_.

Calculated ^a^		Observed (N_2_-matrix) ^b^		Assignment ^c^
ν	Δν	ν	Δν	
3166 (<1)	0.0			ν_asym_ (CH_2_)
3096 (8)	0.0			ν_sym_ (CH_2_)
1497 (1)	0.3	1470.3/1465.3 (2)	<0.5	δ (CH_2_)
1394 (198)	0.3	1387.0/1381.6 (57)	<0.5	ν_asym_ (SO_2_)
1347 (26)	4.2	1326.0/1324.7 (10)	4.4	ω (CH_2_) + ν (N=S)
1287 (303)	11.6	1283.7/1278.0 (100)	10.4	ν (N=S)
1241 (52)	0.8	1224.2/1219.7 (14)	<0.5	τ (CH_2_)
1125 (33)	15.5	1114.6/1113.1 (13)	15.5	ν (CN) + ν_sym_ (SO_2_)
985 (1)	2.6	967.7/955.9 (12)	<0.5	ρ (CH_2_)
854 (20)	9.1	849.4/846.7 (7)	8.8	ν (C–N–S)
712 (81)	0.8	706.4/699.0 (11)	2.3	ν (CCl)
550 (4)	6.0			δ (S–O–N)
502 (53)	2.0	509.3/506.7 (8)	2.2	δ (SO_2_)

^a^ The calculated IR harmonic frequencies (ν, unscaled), ^15^N-isotopic shifts (Δν), and intensities (km mol^−1^, in parentheses) at the B3LYP/6-311++G(3df,3pd) level. Full list of the calculated IR frequencies for all the conformers are given in Table S4. ^b^ The observed band positions (ν), ^15^N-isotopic shifts (Δν), and relative band intensities (in parentheses) in N_2_-matrix. ^c^ Tentative assignment based on the calculated vibrational displacement vectors.

**Table 3:** Calculated and observed IR frequencies (>500 cm^−1^) of CHCl_2_S(O)_2_N.

Calculated ^a^				Observed ^b^		Assignment ^c^
Singlet		Triplet		N_2_-matrix		
ν	Δν	ν	Δν	ν	Δν	
3167 (8)	0.0	3152 (6)	0.0	3027.2 (4)	<0.5	ν(CH)
1409 (139)	2.7	1367 (137)	0.0	1366.5 (100)	<0.5	ν_as__ym_(SO_2_)
1225 (15)	0.0	1230 (11)	0.0	1196.1 (7)	<0.5	ρ(CH)
1213 (5)	0.1	1206 (9)	0.0	1163.7 (4)	<0.5	*ω*(CH)
1048 (74)	10.0	1157 (93)	0.0	1153.2 (32)	<0.5	ν_s__ym_(SO_2_) + *ω*(CH)
978 (13)	11.1	777 (67)	0.2	808.4 (35)	<0.5	ν_as__ym_(CCl_2_)
762 (100)	0.1	764 (24)	0.4	747.7 (16)	<0.5	ν_s__ym_(CCl_2_)
760 (17)	0.6	701 (21)	10.7	718.5 (7)	9.6	ν(SN)
681 (13)	0.4	654 (10)	0.4	675.8 (5)	<0.5	ν(SC)
520 (101)	2.7	507 (82)	1.3	512.6 (50)	<0.5	δ(SO_2_)

^a^ The calculated IR harmonic frequencies (ν, unscaled), ^15^N-isotopic shifts (Δν), and band intensities (km mol^–1^, in parentheses) for the singlet and triplet states at the B3LYP/6-311++G(3df,3pd) level. Full list of the calculated IR frequencies for all the conformers are given in Table S6. ^b^ The observed band positions (ν), ^15^N-isotopic shifts (Δν), and relative intensities (in parentheses) in N_2_-matrix. ^c^ Tentative assignment based on the calculated vibrational displacement vectors for the triplet state.

**Table 4:** Calculated and observed IR frequencies (> 500 cm^–1^) of CHCl_2_NSO_2_.

Calculated ^a^				Observed ^b^		Assignment ^c^
Singlet-I		Singlet-II		N_2_-matrix		
ν	Δν	ν	Δν	ν	Δν	
3151 (2)	0.0	3175 (1)	0.0			ν(CH)
1403 (190)	0.2	1409 (163)	0.0	1410.2/1401.7 (100)	<0.5	ν_asym_(SO_2_)
1340 (7)	1.7	1348 (97)	9.1	1335.0/1323.8 (22)	<0.5	ρ(CH)
1299 (431)	15.0	1278 (326)	7.8	1280.5/1262.7 (98)	16.0	ν(SN) + δ(SO_2_)
1252 (27)	0.0	1243 (25)	0.9	1230.2/1227.1 (7)	<0.5	*ω*(CH)
1129 (32)	15.3	1152 (52)	14.3	1125.4/1117.0 (35)	11.8	ν(CN) + ν_sym_(SO_2_)
890 (44)	12.1	845 (47)	8.1	846.2/834.4 (33)	9.0	ν(CN)
758 (58)	1.4	721 (201)	1.0	783.9/776.9 (56)	<0.5	ν_sym_(CCl_2_)
745 (169)	0.9	714 (18)	2.5	767.2/759.7 (40)	<0.5	ν_asym_(CCl_2_)
526 (70)	1.8	597 (13)	8.5	532.5 (13)	2.2	δ(SO_2_)

^a^ The calculated IR harmonic frequencies (ν, unscaled), ^15^N-isotopic shifts (Δν), and band intensities (km mol^–1^, in parentheses) at the B3LYP/6-311++G(3df,3pd) level. Full list of the calculated IR frequencies for all the conformers are given in Table S4. ^b^ The observed band positions (ν), ^15^N-isotopic shifts (Δν), and relative intensities (in parentheses) in N_2_-matrix. ^c^ Tentative assignment based on the calculated vibrational displacement vectors.

## Supplementary Materials

The following are available online. Calculated vertical transitions, structures, energies and IR frequencies, and Calculated atomic coordinates for all optimized structures.

## References

[1] Gritsan N.P., Falvey D.E., Gudmundsdottir A.D. (2013). Properties of Carbonyl Nitrenes and Related Acyl Nitrenes. Nitrenes and Nitrenium Ions.

[2] Jiang H.L., Lang K., Lu H.J., Wojtas L., Zhang X.P. (2016). Intramolecular radical aziridination of allylic sulfamoyl azides by cobalt(II)-based metalloradical catalysis: Effective construction of strained heterobicyclic structures. Angew. Chem. Int. Ed..

[3] Lu H.J., Li C.Q., Jiang H.L., Lizardi C.L., Zhang X.P. (2014). Chemoselective amination of propargylic C(sp^3^)‒H bonds by cobalt(II)-based metalloradical catalysis. Angew. Chem. Int. Ed..

[4] Liu L.-H., Yan M. (2010). Perfluorophenyl azides: New applications in surface functionalization and nanomaterial synthesis. Acc. Chem. Res..

[5] Lwowski W. (1967). Nitrenes and the decomposition of carbonylazides. Angew. Chem. Int. Ed..

[6] Kundu S., Miceli E., Farquhar E., Pfaff F.F., Kuhlmann U., Hildebrandt P., Braun B., Greco C., Ray K. (2012). Lewis acid trapping of an elusive copper−tosylnitrene intermediate using scandium triflate. J. Am. Chem. Soc..

[7] Gritsan N.P., Likhotvorik I., Tsao M.-L., Celebi N., Platz M.S., Karney W.L., Kemnitz C.R., Borden W.T. (2001). Ring-expansion reaction of cyano-substituted singlet phenyl nitrenes: Theoretical predictions and kinetic results from laser flash photolysis and chemical trapping experiments. J. Am. Chem. Soc..

[8] Kubicki J., Luk H.L., Zhang Y., Vyas S., Peng H.-L., Hadad C.M., Platz M.S. (2012). Direct observation of a sulfonyl azide excited state and its decay processes by ultrafast time-resolved IR Spectroscopy. J. Am. Chem. Soc..

[9] Kuzmin A.V., Neumann C., van Wilderen L.J.G.W., Shainyanb B.A., Bredenbeck J. (2016). Exploring photochemistry of p-bromophenylsulfonyl, p-toylsulfonyl and methylsulfonyl azides by ultrafast UV-Pump-IR-Probe spectroscopy and computations. Phys. Chem. Chem. Phys..

[10] Kubicki J., Zhang Y., Xue J., Luk H.L., Platz M.S. (2012). Ultrafast time resolved studies of the photochemistry of acyl and sulfonyl azides. Phys. Chem. Chem. Phys..

[11] Zeng X.Q., Beckers H., Willner H. (2013). Thermally persistent fluorosulfonyl nitrene and unexpected formation of the fluorosulfonyl radical. J. Am. Chem. Soc..

[12] Zeng X.Q., Beckers H., Willner H., Neuhaus P., Grote D., Sander W. (2015). Photochemistry of matrix isolated (trifluoromethyl)sulfonyl azide, CF_3_SO_2_N_3_. J. Phys. Chem. A.

[13] Zeng X.Q., Beckers H., Neuhaus P., Grote D., Sander W. (2012). Elusive fluoro sulfinyl nitrite, FS(O)NO, produced by photolysis of matrix-isolated FS(O)_2_N. Z. Anorg. Allg. Chem..

[14] Obenhuber A.H., Gianetti T.L., Berrebi X., Bergman R.G., Arnold J. (2014). Reaction of (bisimido)niobium(V) complexes with organic azides: [3+2] cycloaddition and reversible cleavage of β-diketiminato ligands involving nitrene transfer. J. Am. Chem. Soc..

[15] Klima R.F., Gudmundsdottir A.D. (2004). Intermolecular triplet-sensitized photolysis of alkyl azides trapping of triplet alkyl nitrenes. J. Photochem. Photobiol. A..

[16] Hayes J.C., Sheridan R.S. (1990). Infrared spectrum of triplet phenylnitrene. On the origin of didehydroazepine in low-temperature matrices. J. Am. Chem. Soc..

[17] Leyva E., Platz M.S., Persy G., Wirz J. (1986). Photochemistry of phenyl azide: The role of singlet and triplet phenylnitrene as transient intermediates. J. Am. Chem. Soc..

[18] Kubicki J., Zhang Y., Vyas S., Burdzinski G., Luk H.L., Wang J., Xue J., Peng H.-L., Pritchina E.A., Sliwa M. (2011). Photochemistry of 2-naphthoyl azide. An ultrafast time-resolved UV-vis and IR spectroscopic and computational Study. J. Am. Chem. Soc..

[19] Vyas S., Kubicki J., Luk H.L., Zhang Y., Gritsan N.P., Hadad C.M., Platz M.S. (2012). An ultrafast time-resolved infrared and UV-vis spectroscopic and computational study of the photochemistry of acyl azides. J. Phys. Org. Chem..

[20] Zeng X.Q., Beckers H., Willner H., Grote D., Sander W. (2011). The missing link: Triplet fluorocarbonyl nitrene FC(O)N. Chem. Eur. J..

[21] Chuprakov S., Worrell B.T., Selander N., Sit R.K., Fokin V.V. (2014). Stereoselective 1,3-insertions of rhodium(II) azavinyl carbenes. J. Am. Chem. Soc..

[22] McIntosh J.A., Coelho P.S., Farwell C.C., Wang Z.J., Lewis J.C., Brown T.R., Arnold F.H. (2013). Enantioselective intramolecular C‒H amination catalyzed by engineered cytochrome P450 enzymes in vitro and in vivo. Angew. Chem. Int. Ed..

[23] Lu H.J., Jiang H.L., Wojtas L., Zhang X.P. (2010). Selective intramolecular C‒H amination through the metalloradical activation of azides: Synthesis of 1,3-diamines under neutral and nonoxidative conditions. Angew. Chem. Int. Ed..

[24] Lwowski W., Scheiffele E. (1965). Curtius and lossen rearrangements. I. The benzenesulfonyl system. J. Am. Chem. Soc..

[25] Sheridan R.S., Rempala P. (1999). Books of Abstracts. Proceedings of the 217th ACS National Meeting.

[26] Deng G.H., Wu Z., Li D.Q., Linguerri R., Francisco J.S., Zeng X.Q. (2016). Simplest *N*-sulfonylamine HNSO_2_. J. Am. Chem. Soc..

[27] Zeng X.Q., Beckers H., Willner W. (2013). The iminyl radical O_2_SN. Angew. Chem. Int. Ed..

[28] Deng G.H., Dong X.L., Liu Q.F., Li D.Q., Li H.M., Sun Q., Zeng X.Q. (2017). The decomposition of benzenesulfonyl azide: A matrix isolation and computational study. Phys. Chem. Chem. Phys..

[29] Weidner K., Giroult A., Panchaud P., Renaud P. (2010). Efficient carboazidation of alkenes using a radical desulfonylative azide transfer process. J. Am. Chem. Soc..

[30] Wu Z., Wan H.B., Xu J., Lu B., Lu Y., Eckhardt A.K., Schreiner P.R., Xie C.J., Guo H., Zeng X.Q. (2018). The near-UV absorber OSSO and its isomers. Chem. Commun..

[31] Deng G.H., Li D.Q., Wu Z., Li H.M., Bernhardt E., Zeng X.Q. (2016). Methanesulfonyl azide: Molecular structure and photolysis in solid noble gas matrices. J. Phys. Chem. A.

[32] Lu Y., Li H.M., Wan H.B., Liu Q., Deng G.H., Zeng X.Q. (2018). Flash vacuum pyrolysis of sulfamoyl azides and chlorides: Facile gas-hhase generation of transient *N*-sulfonylamines. J. Anal. Appl. Pyrolysis..

[33] Schriver-Mazzuoli L., Chaabouni H., Schriver A. (2003). Infrared spectra of SO_2_ and SO_2_: H_2_O ices at low temperature. J. Mol. Struct..

[34] Allavena M., Rysnik R., White D., Calder V., Mann D.E. (1969). Infrared spectra and geometry of SO_2_ isotopes in solid krypton matrices. J. Chem. Phys..

[35] Hooper N., Beeching L.J., Dyke J.M., Morris A., Ogden J.S. (2002). A study of the thermal decomposition of 2-azidoethanol and 2-azidoethyl acetate by ultraviolet photoelectron spectroscopy and matrix isolation infrared spectroscopy. J. Phys. Chem. A.

[36] Wu Z., Liu Q.F., Li D.Q., Feng R.J., Zeng X.Q. (2017). Flash vacuum pyrolysis of methoxysulfinyl azide: Stepwise decomposition via methoxysulfinyl nitrene. J. Anal. Appl. Pyrolysis..

[37] Zeng X.Q., Bernhardt E., Beckers H., Banert K., Hagedorn M., Liu H.L. (2013). Formyl azide: Properties and solid-state structure. Angew. Chem. Int. Ed..

[38] d’Hendecourt L.B., Grim R.J.A. (1986). Time-dependent chemistry in dense molecular clouds. Astron. Astrophys..

[39] Nonella M., Huber J.R., Ha T.K. (1987). Photolytic preparation and isomerization of HNSO, HOSN, HSNO, and HONS in an argon matrix. An experimental and theoretical study. J. Phys. Chem..

[40] Tobón Y.A., Nieto L.I., Romano R.M., Della Védova C.O., Downs A.J. (2006). Photochemical reaction channels of OCS with Cl_2_, ICl, or IBr isolated together in an argon matrix: Isolation of syn-iodocarbonylsulfenyl bromide. J. Phys. Chem. A.

[41] Ramos L.A., Zeng X.Q., Ulic S.E., Beckers H., Willner H., Della Védova C.O. (2012). Chlorodifluoroacetyl azide, ClF_2_CC(O)N_3_: Preparation, Properties, and Decomposition. J. Org. Chem..

[42] Fridgen T.D., Zhang X.K., Parnis J.M., March R.E. (2000). Isomerization and fragmentation products of CH_2_Cl_2_ and other dihalomethanes in rare-gas matrices: An electron bombardment matrix-isolation FTIR spectroscopic study. J. Phys. Chem. A.

[43] Tsang W. (1990). Mechanisms for the formation and destruction of chlorinated organic products of incomplete combustion. Combust. Sci. Technol..

[44] Yang S.X., Hou G.Y., Dai J.H., Chang C.H., Chang B.C. (2010). Spectroscopic investigation of the multiphoton photolysis reactions of bromomethanes (CHBr_3_, CHBr_2_Cl, CHBrCl_2_, and CH_2_Br_2_) at near-ultraviolet wavelengths. J. Phys. Chem. A.

[45] Carver T.G., Andrews L. (1969). Matrix infrared spectrum and bonding in the dichloromethyl radical. J. Chem. Phys..

[46] Lu Y., Li H.M., Abe M., Bégué D., Wan H.B., Deng G.H., Xu J., Liu K., Zeng X.Q. (2018). Sulfamoyl nitrenes: Singlet or triplet ground state?. Chem. Commun..

[47] Wentrup C. (2018). Carbenes and nitrenes: Recent developments in fundamental chemistry. Angew. Chem. Int. Ed..

[48] Wentrup C. (2017). Flash vacuum pyrolysis of azides, triazoles, and tetrazoles. Chem. Rev..

[49] Wasylenko W.A., Kebede N., Showalter B.M., Matsunaga N., Miceli A.P., Liu Y., Ryzhkov L.R., Hadad C.M., Toscano J.P. (2006). Generation of oxynitrenes and confirmation of their triplet ground states. J. Am. Chem. Soc..

[50] Sun H.L., Zhu B.F., Wu Z., Zeng X.Q., Beckers H., Jenks W.S. (2015). Thermally persistent carbonyl nitrene: FC(O)N. J. Org. Chem..

[51] Li H.M., Wu Z., Li D.Q., Wan H.B., Xu J., Abe M., Zeng X.Q. (2017). Direct observation of methoxycarbonylnitrene. Chem. Commun..

[52] Feng R.J., Lu Y., Deng G.H., Xu J., Wu Z., Li H.M., Liu Q., Kadowaki N., Abe M., Zeng X.Q. (2018). Magnetically bistable nitrenes: Matrix isolation of furoylnitrenes in both singlet and triplet states and triplet 3-furylnitrene. J. Am. Chem. Soc..

[53] Yeh P.-S., Leu G.-H., Lee Y.-P., Chen I.-C. (1995). Photodissociation of HNO_3_ at 193 nm: Near-infrared emission of NO detected by time-resolved fourier transform spectroscopy. J. Chem. Phys..

[54] Tsao M.-L., Hadad C.M., Platz M.S. (2002). A computational study of cyclopropylnitrene. Tetrahedron Lett..

[55] Becke A.D. (1993). Density-functional thermochemistry. III. The role of exact exchange. J. Chem. Phys..

[56] Stratmann R.E., Scuseria G.E., Frisch M.J. (1998). An efficient implementation of time-dependent density-functional theory for the calculation of excitation energies of large molecules. J. Chem. Phys..

[57] Foresman J.B., Head-Gordon M., Pople J.A., Frisch M.J. (1992). Toward a systematic molecular orbital theory for excited states. J. Phys. Chem..

[58] Fukui K. (1981). The path of chemical reactions – The IRC approach. Acc. Chem. Res..

[59] Hratchian H.P., Schlegel H.B. (2005). Using hessian updating to increase the efficiency of a hessian based predictor-corrector reaction path following method. J. Chem. Theory Comput..

[60] Frisch M.J., Trucks G.W., Schlegel H.B., Scuseria G.E., Robb M.A., Cheeseman J.R., Scalmani G., Barone V., Mennucci B., Petersson G.A. (2013). Gaussian, 09.

