# Supplementary material for: Chloro- and Dichloro-methylsulfonyl Nitrenes: Spectroscopic Characterization, Photoisomerization, and Thermal Decomposition
Source: Molecules. 2018 Dec 13;23(12):3312. doi: 10.3390/molecules23123312 (PMC6321523; doi:10.3390/molecules23123312)
Supplement: Supplementary file 1 [file molecules-23-03312-s001.pdf]

Supplementary Materials

Chloro- and Dichloro-methylsulfonyl Nitrenes: Spectroscopic Characterization, Photoisomerization, and Thermal Decomposition

Yang Yang [1], Xianxu Chu [1], Yan Lu [1], Manabu Abe [2,*], and Xiaoqing Zeng [1,*]

1 College of Chemistry, Chemical Engineering and Materials Science, Soochow University, Suzhou, 215123, China; 20174209103@stu.suda.edu.cn (Y.Y.); 20164209104@stu.suda.edu.cn (X.C.); 20164209106@stu.suda.edu.cn (Y.L.); xqzeng@suda.edu.cn (X.Z.)

2 Department of Chemistry, Graduate School of Science, Hiroshima University, 1-3-1 Kagamiyama, Higashi-Hiroshima Hiroshima 739-8526, Japan; mabe@hiroshima-u.ac.jp (M.A.)

* Correspondence: xqzeng@suda.edu.cn (X.Z.); mabe@hiroshima-u.ac.jp (M.A.); Tel.:+86-51265880090 (X.Z.); +81-824247432 (M.A.)

Table of Contents

TD-DFT calculated vertical transitions of $CH_2ClS(O)_2N$ (Table S1)……………………………S2

Calculated energies, IR frequencies for $CH_2ClS(O)_2N$ isomers (Table S2)……………S3

Calculated structures, energies, IR frequencies for $CH_2ClS(O)NO$ isomers (Table S3)……S4

Calculated structures, energies, IR frequencies for CH_2ClNSO_2 and $CHCl_2NSO_2$ (Table S4)……………………………………………………………………………………S5

Calculated structures, energies, IR frequencies for $CH_2ClS(O)NO$ isomers (Table S5)………S6

Calculated energies, IR frequencies for $CHCl_2S(O)_2N$ isomers (Table S6)………………………S7

Calculated energies and atomic coordinates for all optimized structures………………S8–S31

Table S1. Calculated vertical transitions for singlet and triplet $CH_2ClS(O)_2N$ at the TD-B3LYP/6-311++G(3df,3pd) level.

**Singlet-I**		**Singlet-II**		**Singlet-III**		**Triplet-I**		**Triplet-II**	
energy (nm)	oscillator strength	energy (nm)	oscillator strength	energy (nm)	oscillator strength	energy (nm)	oscillator strength	energy (nm)	oscillator strength
514	0.0011	528	0.0008	534	0.0007	509	0.0002	407	0.0003
266	0.0027	272	0.0006	276	0.0013	386	0.0002	390	0.0084
244	0.0005	245	0.0012	252	0.0002	374	0.0004	370	0.0001
238	0.0028	237	0.0052	240	0.0049	356	0.0008	351	0.0241
229	0.0342	227	0.0119	222	0.0011	345	0.0047	339	0.0003
222	0.0016	225	0.0041	218	0.0065	330	0.0008	333	0.0015
202	0.0009	209	0.0013	208	0.0015	323	0.0006	325	0.0001
198	0.0034	203	0.0020	200	0.0044	317	0.0023	297	0.0011
184	0.0146	183	0.0061	186	0.0022	290	0.0027		
182	0.0036	180	0.0017	178	0.0302				

Table S2. Calculated molecular energies (a.u.), IR frequencies and ^{15}N isotopic shifts (cm^{-1}) for all conformers of CH_2ClSO_2N.

Singlet-I		Singlet-II		Singlet-III		Triplet-I		Triplet-II	
–1102.863203 [a]		–1102.862882 [a]		–1102.862522 [a]		–1102.887294 [a]		–1102.885902 [a]	
ν [b]	Δν [c]	ν [b]	Δν [c]	ν [b]	Δν [c]	ν [b]	Δν [c]	ν [b]	Δν [c]
3183 (4)	0.0	3187 (3)	0.0	3158 (3)	0.0	3163 (4)	0.2	3176 (2)	0.0
3098 (5)	0.0	3100 (4)	0.0	3090 (7)	0.0	3086 (9)	0.3	3096 (3)	0.0
1431 (5)	0.0	1434 (4)	0.0	1434 (6)	0.0	1428 (7)	0.4	1434 (4)	0.0
1400 (164)	2.5	1397 (166)	2.4	1387 (159)	2.7	1355 (149)	0.0	1357 (155)	0.0
1268 (13)	0.1	1263 (9)	0.1	1263 (25)	0.4	1259 (11)	0.3	1265 (10)	0.0
1161 (1)	0.0	1149 (3)	0.0	1160 (1)	0.0	1161 (70)	0.1	1158 (83)	0.0
1053 (67)	9.4	1050 (82)	10.7	1059 (79)	9.3	1141 (42)	0.8	1148 (2)	0.0
973 (12)	11.5	972 (15)	9.1	979 (12)	10.0	866 (13)	1.0	875 (2)	0.0
870 (0.1)	0.1	884 (3)	0.9	844 (4)	1.1	780 (18)	0.1	778 (6)	0.2
761 (21)	0.5	768 (23)	0.3	799 (29)	0.2	732 (38)	3.9	699 (15)	11.2
686 (16)	0.9	700 (34)	2.3	681 (30)	2.6	640 (6)	10.2	656 (26)	0.2
515 (83)	3.0	484 (58)	1.8	477 (60)	2.1	492 (45)	0.9	497 (69)	4.0
423 (18)	2.3	432 (20)	2.0	431 (18)	2.9	428 (42)	3.0	463 (19)	2.3
381 (6)	5.1	387 (3)	5.6	374 (6)	4.5	340 (5)	1.2	324 (2)	1.6
301 (1)	0.3	297 (1)	1.4	329 (<1)	0.4	270 (1)	3.3	264 (3)	2.9
277 (<1)	4.2	279 (1)	2.0	247 (1)	3.0	254 (2)	3.2	250 (2)	4.1
164 (2)	1.0	159 (2)	0.6	163 (1)	2.5	155 (1)	0.8	154 (1)	1.0
65 (4)	0.2	63 (4)	0.5	85 (5)	0.2	69 (4)	0.6	61 (2)	0.2

[a] Calculated total energies (Hartree). [b] Calculated harmonic frequencies (cm^{-1},unscaled) and intensities ($km\ mol^{-1}$) in parenthese. [c] Calculated ^{15}N isotopic shifts (cm^{-1}).

Table S3. Calculated molecular structures, energies (a.u.), IR frequencies (cm^{-1}) and ^{15}N isotope shifts (cm^{-1}) for various $CH_2ClS(O)NO$ conformers at the B3LYP/6-311++G(3df,3pd) level.

**Syn-I**		**Syn-II**		**Syn-III**		**Anti-I**		**Anti-II**		**Anti-III**	
−1102.929144 [a]		−1102.929390 [a]		−1102.930412 [a]		−1102.923542 [a]		−1102.924114 [a]		−1102.926645 [a]	
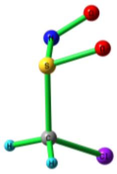		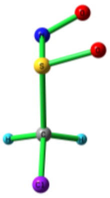		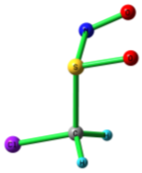		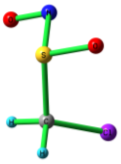		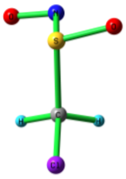		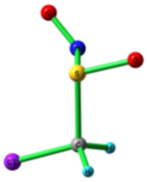	
ν [b]	Δν [c]	ν [b]	Δν [c]	ν [b]	Δν [c]	ν [b]	Δν [c]	ν [b]	Δν [c]	ν [b]	Δν [c]
3151 (<1)	0.0	3157 (<1)	0.0	3160 (1)	0.0	3150 (1)	0.0	3185 (<1)	0.0	3163 (1)	0.0
3077 (5)	0.0	3075 (3)	0.0	3085 (4)	0.0	3077 (5)	0.0	3074 (5)	0.0	3082 (3)	0.0
1845 (530)	32.6	1838 (622)	32.1	1843 (556)	32.2	1784 (543)	31.5	1775 (617)	30.2	1819 (658)	31.6
1426 (5)	0.1	1419 (4)	0.0	1450 (3)	0.0	1431 (7)	0.2	1421 (4)	0.1	1439 (5)	0.0
1237 (12)	0.0	1235 (47)	0.0	1227 (24)	0.0	1232 (16)	0.0	1237 (49)	0.2	1227 (12)	0.0
1160 (54)	0.1	1161 (25)	0.0	1122 (162)	0.0	1172 (151)	0.4	1176 (90)	0.4	1114 (192)	0.0
1125 (92)	0.0	1126 (110)	0.1	1098 (1)	0.0	1132 (53)	0.0	1140 (104)	0.4	1107 (4)	0.1
827 (<1)	0.0	848 (5)	0.0	851 (3)	0.0	829 (<1)	0.0	854 (4)	0.2	857 (4)	0.5
729 (25)	0.3	723 (48)	0.0	744 (35)	0.1	729 (28)	0.0	731 (47)	0.5	740 (29)	0.0
622 (7)	4.3	637 (15)	2.6	693 (5)	0.0	620 (8)	5.6	619 (9)	1.7	710 (9)	0.1
571 (10)	6.2	579 (15)	7.9	601 (18)	10.5	579 (8)	6.3	605 (13)	10.5	606 (16)	12.1
419 (30)	0.8	356 (7)	0.5	319 (17)	1.7	412 (26)	0.4	351 (8)	0.2	321 (3)	0.4
300 (16)	2.6	321 (18)	2.6	306 (15)	2.0	278 (5)	1.3	278 (3)	1.0	254 (3)	2.2
213 (3)	3.2	223 (9)	1.2	240 (5)	2.6	212 (10)	3.0	212 (22)	3.2	213 (16)	0.7
195 (<1)	1.5	190 (<1)	2.8	178 (1)	0.1	165 (4)	0.1	150 (1)	1.5	178 (7)	2.4
141 (<1)	0.8	149 (2)	0.4	145 (1)	0.2	141 (2)	1.2	149 (3)	1.8	146 (6)	0.5
104 (<1)	0.2	99 (<1)	0.3	130 (4)	1.7	92 (<1)	0.5	89 (5)	0.2	77 (5)	0.6
66 (2)	0.3	58 (3)	0.0	61 (2)	0.3	70 (<1)	0.4	48 (1)	0.9	48 (<1)	0.6

[a] Calculated total energies (Hartree). [b] Calculated harmonic frequencies (cm^{-1}, unscaled) and intensities (km mol^{-1}) in parenthese. [c] Calculated ^{15}N isotopic shifts (cm^{-1}).

Table S4. Calculated molecular structures, energies (a.u.), IR frequencies and ^{15}N isotopic shifts (cm^{-1}) for all conformers of CH_2ClNSO_2 and $CHCl_2NSO_2$.

CH_2ClNSO_2		$CHCl_2NSO_2$			
Singlet		Singlet-I		Singlet-II	
–1102.972225 [a]		–11562.602942 [a]		–1562.601260 [a]	
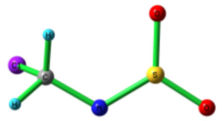		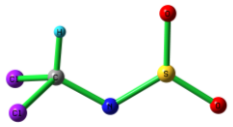		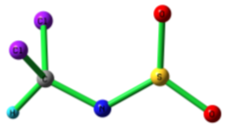	
ν [b]	Δν [c]	ν [b]	Δν [c]	ν [b]	Δν [c]
3166 (<1)	0.0	3151 (2)	0.0	3175 (1)	0.0
3096 (8)	0.0	1403 (190)	0.2	1409 (163)	0.0
1497 (1)	0.3	1340 (7)	1.7	1348 (97)	9.1
1394 (198)	0.3	1299 (431)	15.0	1278 (326)	7.8
1347 (26)	4.2	1252 (27)	0.0	1243 (25)	0.9
1287 (303)	11.6	1129 (32)	15.3	1152 (52)	14.3
1241 (52)	0.8	890 (44)	12.1	845 (47)	8.1
1125 (33)	15.5	758 (58)	1.4	721(201)	1.0
985 (1)	2.6	745 (169)	0.9	714 (18)	2.5
854 (20)	9.1	526 (70)	1.8	597 (13)	8.5
712 (81)	0.8	496 (6)	3.6	494 (50)	1.4
550 (4)	6.0	432 (8)	4.1	458 (12)	5.5
502 (53)	2.0	373 (6)	4.7	357 (10)	0.7
441 (17)	3.2	356 (<1)	0.2	343 (6)	0.1
349 (3)	3.4	235 (1)	0.6	247 (1)	0.6
235 (6)	0.5	158 (1)	0.9	131 (<1)	0.3
118 (<1)	0.7	104 (<1)	0.2	102 (<1)	0.4
48 (1)	0.0	33 (<1)	0.1	61 (<1)	0.0

[a] Calculated total energies (Hartree). [b] Calculated harmonic IR frequencies (cm^{-1},unscaled) and intensities (km mol^{-1}) in parenthese with the B3LYP/6-311++G3df,3pd) basis set. [c] Calculated ^{15}N isotopic shifts (cm^{-1}).

Table S5. Calculated molecular structures, energies (a.u.), IR frequencies (cm^{-1}) and ^{15}N isotope shifts (cm^{-1}) for various $CHCl_2S(O)NO$ conformers at the B3LYP/6-311++G(3df,3pd) level.

**Syn-I**		**Syn-II**		**Syn-III**		**Anti-I**		**Anti-II**		**Anti-III**	
–1562.560396 [a]		–1562.558278 [a]		–1562.560284 [a]		–1562.552606 [a]		–1562.554322 [a]		–1562.556151 [a]	
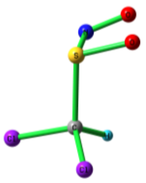		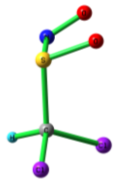		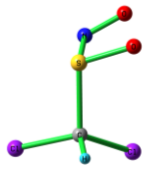		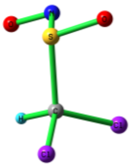		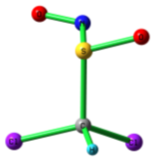		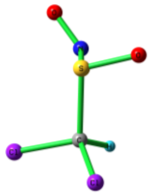	
ν [b]	Δν [c]	ν [b]	Δν [c]	ν [b]	Δν [c]	ν [b]	Δν [c]	ν [b]	Δν [c]	ν [b]	Δν [c]
3138 (1)	0.0	3125 (2)	0.0	3142 (2)	0.0	3124 (2)	0.0	3137 (3)	0.0	3132 (1)	0.0
1866 (645)	32.8	1864 (625)	32.7	1880 (571)	33.0	1789 (623)	31.5	1835 (667)	32.3	1831 (763)	32.3
1237 (29)	0.0	1219 (13)	0.0	1237 (12)	0.0	1228 (17)	0.0	1230 (15)	0.0	1234 (26)	0.2
1157 (34)	0.0	1182 (17)	0.0	1163 (18)	0.0	1187 (58)	0.0	1168 (54)	0.0	1164 (33)	0.1
1128 (126)	0.0	1140 (122)	0.1	1131 (144)	0.0	1161 (143)	0.1	1141 (159)	0.0	1125 (156)	0.0
748 (107)	0.0	751 (49)	0.0	758 (106)	0.0	745 (65)	0.0	750 (111)	0.0	757 (100)	0.0
718 (22)	0.1	711 (78)	0.0	715 (13)	0.2	721 (64)	0.0	714 (11)	0.1	727 (15)	1.3
668 (20)	0.0	620 (6)	5.8	619 (7)	0.0	609 (16)	9.6	634 (10)	4.0	705 (44)	2.0
590 (21)	10.2	544 (12)	4.6	586 (16)	10.1	580 (<1)	2.3	592 (17)	7.3	565 (9)	9.8
355 (7)	1.2	448 (26)	0.2	403 (31)	1.0	441 (24)	0.1	365 (20)	1.2	342 (3)	0.2
333 (2)	0.4	343 (13)	2.4	326 (1)	0.0	308 (3)	0.9	332 (2)	0.2	327 (2)	0.1
285 (22)	2.1	284 (3)	0.5	284 (12)	2.5	268 (1)	0.3	250 (5)	0.6	248 (3)	0.3
228 (2)	0.9	214 (6)	2.0	228 (2)	1.1	208 (18)	0.8	196 (9)	1.5	205 (21)	0.7
217 (3)	1.6	190 (2)	1.4	194 (<1)	2.1	171 (1)	0.4	162 (2)	2.0	182 (7)	1.6
168 (3)	1.0	159 (<1)	0.9	176 (1)	0.2	143 (<1)	3.6	152 (1)	0.8	162 (2)	1.7
132 (2)	1.3	134 (3)	0.9	134 (3)	1.1	134 (2)	0.4	149 (4)	1.9	109 (<1)	1.8
93 (<1)	0.4	99 (<1)	0.4	102 (<1)	0.7	80 (<1)	0.0	66 (<1)	0.0	64 (<1)	0.0
47 (<1)	0.3	35 (<1)	0.1	53 (1)	0.3	41 (<1)	0.2	39 (<1)	0.0	46 (<1)	0.0

[a] Calculated total energies (Hartree). [b] Calculated harmonic frequencies (cm^{-1},unscaled) and intensities (km mol^{-1}) in parenthese. [c] Calculated ^{15}N isotopic shifts (cm^{-1}).

Table S6. Calculated molecular energies (a.u.), IR frequencies and ^{15}N isotopic shifts (cm^{-1}) for all conformers of $CHCl_2SO_2N$.

**Singlet-I**		**Singlet-II**		**Singlet-III**		**Triplet-I**		**Triplet-II**	
–1562.487491 [a]		–1562.487065 [a]		–1562.489744 [a]		–1562.512925 [a]		–1562.512157 [a]	
ν [b]	Δν [c]	ν [b]	Δν [c]	ν [b]	Δν [c]	ν [b]	Δν [c]	ν [b]	Δν [c]
3145 (6)	0	3147 (4)	0	3167 (8)	0	3152 (6)	0	3124 (9)	0
1397 (147)	2.7	1397 (145)	2.6	1409 (139)	2.5	1367 (137)	0	1362 (141)	0
1223 (16)	0	1225 (13)	0	1225 (15)	0	1230 (11)	0	1213 (14)	0
1199 (4)	0.4	1217 (6)	0.4	1213 (5)	0.1	1206 (9)	0	1210 (12)	0
1055 (84)	9.1	1056 (65)	8.8	1048 (74)	10.0	1157 (93)	0	1145 (98)	0
984 (10)	10.9	982 (9)	11.8	978 (13)	11.1	777 (67)	0.2	780 (96)	0
797 (80)	0.2	792 (86)	0	762 (100)	0.1	764 (24)	0.4	776 (13)	2.8
738 (37)	0.5	736 (22)	0.5	760 (17)	0.6	701 (21)	10.7	733 (28)	2.4
668 (27)	2.4	650 (9)	0.9	681 (13)	0.4	654 (10)	0.4	615 (9)	9.6
497 (73)	1.8	518 (102)	2.3	520 (101)	2.7	507 (82)	1.3	507 (60)	1.6
424 (27)	2.4	439 (18)	2.8	444 (18)	1.4	472 (26)	4.9	405 (50)	2.6
388 (4)	5.0	376 (8)	5.0	387 (3)	5.9	351 (4)	0.5	353 (7)	1.4
347 (1)	0.3	349 (1)	0.4	339 (3)	0.8	298 (4)	3.1	337 (1)	0.3
291 (1)	1.0	279 (<1)	2.3	277 (<1)	2.3	258 (1)	3.0	260 (<1)	3.2
242 (<1)	0.7	242 (<1)	1.7	222 (1)	1.6	225 (<1)	1.9	236 (<1)	0.5
174 (<1)	2.8	174 (<1)	0.2	186 (1)	0.1	182 (<1)	0	165 (1)	0
163 (1)	0.6	166 (1)	1.4	141 (1)	1.0	141 (1)	1.4	159 (1)	3.5
39 (<1)	0.3	43 (<1)	0.2	57 (<1)	0.4	47 (<1)	0.1	34 (<1)	0.3

[a] Calculated total energies (Hartree), [b] Calculated harmonic frequencies (cm^{-1},unscaled) and intensities (km mol^{-1}) in parenthese. [c] Calculated ^{15}N isotopic shifts (cm^{-1}).

Calculated Atomic Coordinates (in Angstroms) and Energies (in Hatrees) for All Opitimized Structures at B3LYP/6-311++G(3df,3pd) Level of Theory

$CH_2ClSO_2N_3$-I

S	-0.15943700	0.80368200	-0.02639700
O	0.43513900	1.57554100	-1.06395100
O	-1.04356500	1.37079800	0.95105200
C	1.13083200	-0.04061900	0.92011500
H	0.63797800	-0.69768900	1.62978300
H	1.65671500	0.75693300	1.43923800
Cl	2.26580700	-0.95821600	-0.07221400
N	-0.94487400	-0.48982900	-0.82409200
N	-1.93935800	-0.94321100	-0.23772100
N	-2.85576900	-1.41774500	0.19945200

Zero-point correction=	0.052138
Thermal correction to Energy=	0.060292
Thermal correction to Enthalpy=	0.061236
Thermal correction to Gibbs Free Energy=	0.017422
Sum of electronic and zero-point Energies=	-1212.442046
Sum of electronic and thermal Energies=	-1212.433892
Sum of electronic and thermal Enthalpies=	-1212.432947
Sum of electronic and thermal Free Energies=	-1212.476762

$CH_2ClSO_2N_3$-II

S	-0.10659900	0.53322600	0.08654300
O	0.31548900	1.81623500	-0.36581600
O	-0.36956900	0.24931000	1.46490800
C	1.00084300	-0.72689200	-0.59488700
H	1.02936400	-0.58032500	-1.66956200
H	0.59591800	-1.69848900	-0.33041200
Cl	2.62832300	-0.56110000	0.07771300
N	-1.49784500	0.14397500	-0.84997200
N	-2.39801900	-0.44536000	-0.23333500
N	-3.27179600	-0.96677400	0.23627100

Zero-point correction=	0.052161
Thermal correction to Energy=	0.060362
Thermal correction to Enthalpy=	0.061306
Thermal correction to Gibbs Free Energy=	0.017332
Sum of electronic and zero-point Energies=	-1212.441494
Sum of electronic and thermal Energies=	-1212.433293
Sum of electronic and thermal Enthalpies=	-1212.432349

Sum of electronic and thermal Free Energies= -1212.476323

$CH_2ClSO_2N_3$-III

S	-0.22498400	0.93358700	0.10760500
O	-0.44422500	2.24042800	-0.42437900
O	-0.44087500	0.61912800	1.48681400
C	1.46002200	0.49795000	-0.37474500
H	2.10067300	1.18971500	0.16586200
N	-1.14167600	-0.12171700	-0.87320100
N	-1.61472300	-1.11184400	-0.29194000
N	-2.10310700	-2.02421600	0.13497900
Cl	1.90010500	-1.16691700	0.03051500
H	1.53449900	0.65077000	-1.44618000

Zero-point correction= 0.052202
Thermal correction to Energy= 0.060312
Thermal correction to Enthalpy= 0.061256
Thermal correction to Gibbs Free Energy= 0.017867
Sum of electronic and zero-point Energies= -1212.444026
Sum of electronic and thermal Energies= -1212.435917
Sum of electronic and thermal Enthalpies= -1212.434972
Sum of electronic and thermal Free Energies= -1212.478362

$CH_2ClSO_2N_3$-I-TS1

S	-0.00319200	0.89068700	0.01236700
O	0.66033000	1.45347000	-1.16463800
O	-0.59409400	1.70376100	1.03133100
C	1.17666900	-0.14204500	0.91481800
H	0.62114300	-0.64901300	1.69799000
H	1.89294300	0.54830600	1.35277600
Cl	2.02175600	-1.32413100	-0.08780000
N	-0.91181500	0.06561900	-0.99847900
N	-2.26171000	-0.87850700	0.03101800
N	-3.17258400	-1.47934700	0.08481800

Zero-point correction= 0.047881
Thermal correction to Energy= 0.056818
Thermal correction to Enthalpy= 0.057762
Thermal correction to Gibbs Free Energy= 0.012134
Sum of electronic and zero-point Energies= -1212.384578
Sum of electronic and thermal Energies= -1212.375640
Sum of electronic and thermal Enthalpies= -1212.374696

Sum of electronic and thermal Free Energies= -1212.420325

CH_2ClSO_2N-singlet-I

S	-0.79975100	0.18422000	-0.08539900
O	-1.24999200	-0.71240600	1.05368200
O	-0.65464400	1.59547300	0.03303300
C	0.72441600	-0.52444300	-0.73642900
H	0.59260000	-1.59990700	-0.70003000
H	0.82438000	-0.16833500	-1.75845100
Cl	2.15624100	-0.04917100	0.19215000
N	-2.05521000	-0.60875100	-0.53097500

Zero-point correction= 0.041054
Thermal correction to Energy= 0.047388
Thermal correction to Enthalpy= 0.048332
Thermal correction to Gibbs Free Energy= 0.009681
Sum of electronic and zero-point Energies= -1102.831830
Sum of electronic and thermal Energies= -1102.825496
Sum of electronic and thermal Enthalpies= -1102.824552
Sum of electronic and thermal Free Energies= -1102.863203

CH_2ClSO_2N-singlet-II

S	0.76306200	0.17773800	0.01756000
O	1.89500600	-0.65533700	-0.55766700
O	0.71202200	1.59920200	-0.05870400
C	-0.73197800	-0.49743500	-0.73567000
H	-0.61105500	-1.57455100	-0.74669100
N	1.36872200	-0.68788800	1.15134200
Cl	-2.16648600	-0.05519300	0.20515100
H	-0.81308800	-0.08206200	-1.73625200

Zero-point correction= 0.041038
Thermal correction to Energy= 0.047385
Thermal correction to Enthalpy= 0.048330
Thermal correction to Gibbs Free Energy= 0.009616
Sum of electronic and zero-point Energies= -1102.831460
Sum of electronic and thermal Energies= -1102.825113
Sum of electronic and thermal Enthalpies= -1102.824169
Sum of electronic and thermal Free Energies= -1102.862882

CH_2ClSO_2N-singlet-III

S	-0.86723400	0.08210700	-0.06039500
O	-0.82987500	-1.08877600	0.89983100
O	-1.84845500	1.11826200	-0.00214000
C	0.71752800	0.95123000	0.03250000
N	-0.92578800	-1.22710200	-0.88206800
H	0.69760300	1.50570400	0.96740800
Cl	2.12069800	-0.10664800	-0.02325700
H	0.72826300	1.64006300	-0.80776800

Zero-point correction=	0.040933
Thermal correction to Energy=	0.047260
Thermal correction to Enthalpy=	0.048204
Thermal correction to Gibbs Free Energy=	0.009735
Sum of electronic and zero-point Energies=	-1102.831323
Sum of electronic and thermal Energies=	-1102.824996
Sum of electronic and thermal Enthalpies=	-1102.824052
Sum of electronic and thermal Free Energies=	-1102.862522

CH_2ClSO_2N-singlet-III-TS2

S	-0.89611700	-0.10909800	-0.04421900
O	-0.55268500	-1.32835700	-0.71716100
O	-1.62603800	-0.22008900	1.20434700
C	0.72890600	0.90263600	0.12921000
H	0.79197600	1.66317800	-0.64254600
H	0.57671700	1.35065100	1.10784100
Cl	2.12945700	-0.13221800	0.07759100
N	-1.45360600	1.13588400	-0.82136900

Zero-point correction=	0.038538
Thermal correction to Energy=	0.044831
Thermal correction to Enthalpy=	0.045776
Thermal correction to Gibbs Free Energy=	0.007125
Sum of electronic and zero-point Energies=	-1102.793862
Sum of electronic and thermal Energies=	-1102.787569
Sum of electronic and thermal Enthalpies=	-1102.786625
Sum of electronic and thermal Free Energies=	-1102.825275

CH_2ClSO_2N-singlet-III-TS3

S	0.86481600	-0.23875700	-0.30317700
O	0.67698200	1.32541500	0.75191100
O	1.80831100	-1.25928300	0.09617700
C	-0.79632000	-0.79185300	0.46883200

N	1.32460900	1.28384400	-0.48111000
H	-0.70427200	-0.66417100	1.53989200
Cl	-2.15713500	0.09132100	-0.18153300
H	-0.83817500	-1.83302200	0.16708500

Zero-point correction= 0.038294
Thermal correction to Energy= 0.044747
Thermal correction to Enthalpy= 0.045691
Thermal correction to Gibbs Free Energy= 0.006793
Sum of electronic and zero-point Energies= -1102.787030
Sum of electronic and thermal Energies= -1102.780576
Sum of electronic and thermal Enthalpies= -1102.779632
Sum of electronic and thermal Free Energies= -1102.818531

CH_2ClSO_2N-triplet-I

S	0.86067500	-0.03430100	-0.06401500
O	1.92715300	0.93027500	-0.07780000
O	0.82344100	-1.12196300	-0.99709300
C	-0.67830300	0.91156200	-0.12092000
H	-0.64368400	1.63849700	0.68479300
H	-0.65908000	1.41007500	-1.08809300
Cl	-2.12766400	-0.09002800	0.01948800
N	0.82390200	-0.70073500	1.48870000

Zero-point correction= 0.040159
Thermal correction to Energy= 0.046697
Thermal correction to Enthalpy= 0.047641
Thermal correction to Gibbs Free Energy= 0.007579
Sum of electronic and zero-point Energies= -1102.854714
Sum of electronic and thermal Energies= -1102.848176
Sum of electronic and thermal Enthalpies= -1102.847232
Sum of electronic and thermal Free Energies= -1102.887294

CH_2ClSO_2N-triplet-II

S	-0.80898000	-0.10926300	0.00007500
O	-0.91702400	-0.80122700	1.25242000
O	-0.91662900	-0.80338300	-1.25111500
C	0.68311100	0.92633300	-0.00046900
H	0.66673000	1.53325200	0.89941900
N	-1.98209900	1.10260100	-0.00115600
Cl	2.12090300	-0.10334600	0.00004000
H	0.66685300	1.53251200	-0.90082500

Zero-point correction=	0.040237
Thermal correction to Energy=	0.046794
Thermal correction to Enthalpy=	0.047739
Thermal correction to Gibbs Free Energy=	0.007543
Sum of electronic and zero-point Energies=	-1102.853208
Sum of electronic and thermal Energies=	-1102.846651
Sum of electronic and thermal Enthalpies=	-1102.845707
Sum of electronic and thermal Free Energies=	-1102.885902

CH_2ClNSO_2

S	1.24303700	-0.00026900	-0.00533100
O	2.36814600	-0.74345800	-0.46985200
O	1.27895300	1.43265200	0.05096100
C	-1.14761800	-0.11081900	0.88125800
H	-1.61455800	-0.72396900	1.64352600
H	-0.96697800	0.89640300	1.24394300
N	0.04254500	-0.80157000	0.43585700
Cl	-2.34682100	0.03495400	-0.45821100

Zero-point correction=	0.043186
Thermal correction to Energy=	0.049390
Thermal correction to Enthalpy=	0.050334
Thermal correction to Gibbs Free Energy=	0.011312
Sum of electronic and zero-point Energies=	-1102.940352
Sum of electronic and thermal Energies=	-1102.934147
Sum of electronic and thermal Enthalpies=	-1102.933203
Sum of electronic and thermal Free Energies=	-1102.972225

CH_2ClNSO_2-TS4

S	1.40660500	-0.02083300	-0.00471800
O	0.71627400	-1.26103800	0.25984600
O	2.83887000	-0.06387900	-0.09427600
C	-1.76820900	1.02425900	0.27422100
N	0.70067400	1.28034700	-0.17605100
Cl	-2.51310800	-0.39432500	-0.16467800
H	-0.56117600	1.31465500	-0.19820900
H	-1.95828500	1.21353600	1.33569700

Zero-point correction=	0.033120
Thermal correction to Energy=	0.040302
Thermal correction to Enthalpy=	0.041246

Thermal correction to Gibbs Free Energy= -0.000911
Sum of electronic and zero-point Energies= -1102.820195
Sum of electronic and thermal Energies= -1102.813013
Sum of electronic and thermal Enthalpies= -1102.812069
Sum of electronic and thermal Free Energies= -1102.854226

$CH_2ClS(O)NO$-syn-I

S	-0.76751100	-0.94131300	-0.30162800
O	-1.02768700	-0.91260500	1.15320000
C	1.03379000	-0.77484800	-0.62034600
H	1.13950400	-0.59479100	-1.68844100
H	1.49953400	-1.71651900	-0.34448700
Cl	1.84723100	0.52618200	0.28601700
N	-1.08914100	1.12099600	-0.74787500
O	-1.51488200	1.56627200	0.21603700

Zero-point correction= 0.039222
Thermal correction to Energy= 0.046618
Thermal correction to Enthalpy= 0.047562
Thermal correction to Gibbs Free Energy= 0.006290
Sum of electronic and zero-point Energies= -1102.896212
Sum of electronic and thermal Energies= -1102.888815
Sum of electronic and thermal Enthalpies= -1102.887871
Sum of electronic and thermal Free Energies= -1102.929144

$CH_2ClS(O)NO$-syn-II

S	-0.39131300	0.49140200	0.57889300
O	-0.79080500	1.43821100	-0.48477500
C	0.77012200	-0.72963300	-0.13930700
H	0.71980700	-1.63721200	0.45745400
N	-1.96131400	-0.94645100	0.28761100
O	-2.64955600	-0.45176100	-0.48333800
Cl	2.45221300	-0.12960300	-0.11648600
H	0.48493600	-0.91061100	-1.17201600

Zero-point correction= 0.039190
Thermal correction to Energy= 0.046614
Thermal correction to Enthalpy= 0.047559
Thermal correction to Gibbs Free Energy= 0.006068
Sum of electronic and zero-point Energies= -1102.896268
Sum of electronic and thermal Energies= -1102.888844
Sum of electronic and thermal Enthalpies= -1102.887900

Sum of electronic and thermal Free Energies= -1102.929390

$CH_2ClS(O)NO$-syn-III

S	0.50276200	0.70119200	-0.50387300
O	1.63609900	1.28693100	0.25198600
C	-0.87652500	0.49089500	0.70369000
H	-1.19554600	1.48334200	1.00675200
N	1.03860400	-1.35737900	-0.22156400
O	2.06197700	-1.30444000	0.28843900
Cl	-2.22993000	-0.34855400	-0.08834500
H	-0.53553300	-0.08064600	1.56248100

Zero-point correction= 0.039323
Thermal correction to Energy= 0.046707
Thermal correction to Enthalpy= 0.047651
Thermal correction to Gibbs Free Energy= 0.006351
Sum of electronic and zero-point Energies= -1102.897439
Sum of electronic and thermal Energies= -1102.890055
Sum of electronic and thermal Enthalpies= -1102.889111
Sum of electronic and thermal Free Energies= -1102.930412

$CH_2ClS(O)NO$-anti-I

S	0.79189900	-0.84283300	0.34423100
O	0.44777500	-1.83251700	-0.68978000
C	-0.74572300	-0.05662900	0.96977700
H	-0.44061500	0.80506300	1.56047900
H	-1.24752600	-0.78743600	1.59752300
Cl	-1.87327600	0.45397500	-0.30986500
N	1.32967600	0.92609800	-0.64123600
O	1.55598300	1.78341900	0.09878000

Zero-point correction= 0.038990
Thermal correction to Energy= 0.046481
Thermal correction to Enthalpy= 0.047425
Thermal correction to Gibbs Free Energy= 0.005809
Sum of electronic and zero-point Energies= -1102.890361
Sum of electronic and thermal Energies= -1102.882869
Sum of electronic and thermal Enthalpies= -1102.881925
Sum of electronic and thermal Free Energies= -1102.923542

$CH_2ClS(O)NO$-anti-I

S	0.44074600	0.64575000	-0.42524600
O	0.28774100	1.89074900	0.34516500
C	-0.59562200	-0.64530100	0.37160500
H	-0.26627400	-1.61589700	0.00934000
N	2.11044600	-0.30390400	0.39519600
O	2.43958400	-1.24698500	-0.18858000
Cl	-2.31246900	-0.43019400	-0.05315500
H	-0.49167900	-0.55378300	1.44955000

Zero-point correction=	0.038900
Thermal correction to Energy=	0.046495
Thermal correction to Enthalpy=	0.047440
Thermal correction to Gibbs Free Energy=	0.005205
Sum of electronic and zero-point Energies=	-1102.890419
Sum of electronic and thermal Energies=	-1102.882823
Sum of electronic and thermal Enthalpies=	-1102.881879
Sum of electronic and thermal Free Energies=	-1102.924114

CHClNH-syn

C	0.00000000	0.75957700	0.00000000
N	1.21388600	1.04892100	0.00000000
H	1.35240800	2.05836400	0.00000000
Cl	-0.52921300	-0.90545000	0.00000000
H	-0.85299400	1.43437200	0.00000000

Zero-point correction=	0.031353
Thermal correction to Energy=	0.034698
Thermal correction to Enthalpy=	0.035643
Thermal correction to Gibbs Free Energy=	0.006102
Sum of electronic and zero-point Energies=	-554.275540
Sum of electronic and thermal Energies=	-554.272194
Sum of electronic and thermal Enthalpies=	-554.271250
Sum of electronic and thermal Free Energies=	-554.300790

CHClNH-anti

C	0.64110600	0.47844700	0.00000000
H	0.67580600	1.56132500	0.00000000
N	1.66196000	-0.23264600	0.00000000
H	1.47367200	-1.23326200	0.00000000
Cl	-1.03704900	-0.09236600	0.00000000

Zero-point correction=	0.031545

Thermal correction to Energy=	0.034914
Thermal correction to Enthalpy=	0.035858
Thermal correction to Gibbs Free Energy=	0.006213
Sum of electronic and zero-point Energies=	-554.279121
Sum of electronic and thermal Energies=	-554.275751
Sum of electronic and thermal Enthalpies=	-554.274807
Sum of electronic and thermal Free Energies=	-554.304452

CH_2ClN-singlet

C	-0.56623400	0.93283600	0.00000000
H	-1.13521100	1.12125400	0.92122000
H	-1.13521100	1.12125400	-0.92122000
Cl	0.00000000	-0.82241100	0.00000000
N	0.80968900	0.87735100	0.00000000

Zero-point correction=	0.028831
Thermal correction to Energy=	0.032296
Thermal correction to Enthalpy=	0.033240
Thermal correction to Gibbs Free Energy=	0.003521
Sum of electronic and zero-point Energies=	-554.136235
Sum of electronic and thermal Energies=	-554.132771
Sum of electronic and thermal Enthalpies=	-554.131827
Sum of electronic and thermal Free Energies=	-554.161546

CH_2Cl

C	0.00000000	0.00000000	-1.11368200
H	0.00000000	-0.95126300	-1.61552300
H	0.00000000	0.95126300	-1.61552300
Cl	0.00000000	0.00000000	0.58312600

Zero-point correction=	0.022737
Thermal correction to Energy=	0.026200
Thermal correction to Enthalpy=	0.027144
Thermal correction to Gibbs Free Energy=	-0.000839
Sum of electronic and zero-point Energies=	-499.468085
Sum of electronic and thermal Energies=	-499.464622
Sum of electronic and thermal Enthalpies=	-499.463678
Sum of electronic and thermal Free Energies=	-499.491661

$CHClSO_2N_3$-I

S	-0.24158400	-0.93793900	0.05236500

O	-0.47366100	-0.80635000	1.45574600
O	0.16197000	-2.15944400	-0.55946200
C	0.96802000	0.33864200	-0.51006100
H	0.99937000	0.26878300	-1.59144000
Cl	2.55451600	-0.07030800	0.14134000
Cl	0.43706400	1.96236200	-0.05476300
N	-1.61586000	-0.43416300	-0.82121800
N	-2.44652100	0.22901400	-0.17696700
N	-3.26697200	0.81483900	0.30845600

Zero-point correction= 0.042639
Thermal correction to Energy= 0.051870
Thermal correction to Enthalpy= 0.052814
Thermal correction to Gibbs Free Energy= 0.006337
Sum of electronic and zero-point Energies= -1672.067021
Sum of electronic and thermal Energies= -1672.057790
Sum of electronic and thermal Enthalpies= -1672.056846
Sum of electronic and thermal Free Energies= -1672.103324

$CHCl_2SO_2N_3$-I-TS1

S	-0.12926900	-0.95473700	0.12740000
O	-0.15330800	-0.88036500	1.54925800
O	0.28851300	-2.14694400	-0.61568900
C	0.94895500	0.39056500	-0.51219200
H	0.92241200	0.30559400	-1.59191000
Cl	2.60569700	0.13729400	0.04469700
Cl	0.30568000	1.97353200	-0.04719800
N	-1.46772700	-1.00947800	-0.72716500
N	-2.71108900	0.34170800	-0.02344100
N	-3.69588200	0.80509100	0.06498100

Zero-point correction= 0.038435
Thermal correction to Energy= 0.048500
Thermal correction to Enthalpy= 0.049444
Thermal correction to Gibbs Free Energy= 0.000768
Sum of electronic and zero-point Energies= -1672.010062
Sum of electronic and thermal Energies= -1671.999997
Sum of electronic and thermal Enthalpies= -1671.999053
Sum of electronic and thermal Free Energies= -1672.047729

$CHClSO_2N_3$-II

S	0.42558400	-0.57454700	0.48792200
O	0.87753500	-1.70685500	-0.26283500
O	-0.01029700	-0.65875200	1.83829000
C	-0.88420200	0.24169000	-0.52997800
Cl	-2.24536300	-0.87713400	-0.65876400
N	1.62923100	0.63778100	0.46194300
N	2.53438100	0.46296200	-0.36980400
N	3.40474000	0.39804600	-1.07196600
H	-0.46224700	0.38472000	-1.51878100
Cl	-1.34041700	1.80603000	0.13800100

Zero-point correction= 0.042543
Thermal correction to Energy= 0.051833
Thermal correction to Enthalpy= 0.052777
Thermal correction to Gibbs Free Energy= 0.005802
Sum of electronic and zero-point Energies= -1672.064990
Sum of electronic and thermal Energies= -1672.055700
Sum of electronic and thermal Enthalpies= -1672.054756
Sum of electronic and thermal Free Energies= -1672.101730

$CHClSO_2N_3$-III

S	-0.37673200	-0.91814500	0.40654800
O	-1.17159200	-0.57021500	1.54495000
O	0.05826600	-2.25087300	0.15650100
C	1.13905800	0.13655200	0.46109800
Cl	0.70402600	1.84255900	0.62816200
N	-1.13228700	-0.39834200	-1.02108400
N	-2.05325100	0.42345100	-0.87209800
N	-2.90951300	1.14347000	-0.86021100
H	1.64723400	-0.18101900	1.36687300
Cl	2.18527400	-0.16957700	-0.92087300

Zero-point correction= 0.042589
Thermal correction to Energy= 0.051811
Thermal correction to Enthalpy= 0.052755
Thermal correction to Gibbs Free Energy= 0.006235
Sum of electronic and zero-point Energies= -1672.066614
Sum of electronic and thermal Energies= -1672.057392
Sum of electronic and thermal Enthalpies= -1672.056448
Sum of electronic and thermal Free Energies= -1672.102968

$CHCl_2SO_2N$-singlet-I

S	1.08861600	-0.19622500	-0.04355400
O	1.73849600	1.16242500	-0.17020500
O	1.55752200	-1.34985500	-0.73643200
C	-0.68880700	-0.03495800	-0.51198400
H	-0.73677800	-0.19309000	-1.58409100
Cl	-1.54803300	-1.36485300	0.28898000
Cl	-1.33859300	1.54180400	-0.11517200
N	1.45089500	0.29053100	1.37874700

Zero-point correction=	0.031395
Thermal correction to Energy=	0.038843
Thermal correction to Enthalpy=	0.039787
Thermal correction to Gibbs Free Energy=	-0.002238
Sum of electronic and zero-point Energies=	-1562.453858
Sum of electronic and thermal Energies=	-1562.446411
Sum of electronic and thermal Enthalpies=	-1562.445467
Sum of electronic and thermal Free Energies=	-1562.487491

$CHCl_2SO_2N$-singlet-II

S	-1.11689000	-0.09697300	-0.14552800
O	-1.25459300	0.22706800	1.32638500
O	-1.60288700	-1.28514900	-0.76485500
C	0.69075300	-0.05021800	-0.53397300
Cl	1.39857300	1.50529600	-0.14336200
N	-1.84941300	1.25754500	-0.01212900
Cl	1.47013200	-1.40295600	0.30376400
H	0.76348600	-0.22505600	-1.60188200

Zero-point correction=	0.031426
Thermal correction to Energy=	0.038868
Thermal correction to Enthalpy=	0.039812
Thermal correction to Gibbs Free Energy=	-0.002130
Sum of electronic and zero-point Energies=	-1562.453508
Sum of electronic and thermal Energies=	-1562.446066
Sum of electronic and thermal Enthalpies=	-1562.445122
Sum of electronic and thermal Free Energies=	-1562.487065

$CHCl_2SO_2N$-singlet-III

S	-1.06455900	-0.05809500	0.18713200
O	-1.87123300	0.86898800	-0.69971700
O	-1.00159700	0.04780000	1.60307900

C	0.62829900	-0.00319000	-0.51023700
N	-2.02393900	-0.91699800	-0.67289600
Cl	1.45564400	1.47648800	-0.02362800
Cl	1.48080800	-1.47424400	-0.02719900
H	0.49367400	-0.00481400	-1.58525100

Zero-point correction=	0.031492
Thermal correction to Energy=	0.038945
Thermal correction to Enthalpy=	0.039889
Thermal correction to Gibbs Free Energy=	-0.001965
Sum of electronic and zero-point Energies=	-1562.456286
Sum of electronic and thermal Energies=	-1562.448834
Sum of electronic and thermal Enthalpies=	-1562.447889
Sum of electronic and thermal Free Energies=	-1562.489744

$CHCl_2SO_2N$-singlet-III-TS2

S	-1.29365700	0.07779100	0.06398200
O	-1.54665000	1.45642900	-0.27019800
O	-1.87138400	-0.94482000	-0.78574300
N	-1.17219000	-0.36620200	1.52588200
C	0.75814100	-0.00819700	-0.45528900
Cl	1.37123700	-1.53963400	0.00607300
Cl	1.63937900	1.37762500	0.05216900
H	0.51881100	0.02921700	-1.51574500

Zero-point correction=	0.028590
Thermal correction to Energy=	0.036244
Thermal correction to Enthalpy=	0.037189
Thermal correction to Gibbs Free Energy=	-0.005201
Sum of electronic and zero-point Energies=	-1562.418512
Sum of electronic and thermal Energies=	-1562.410859
Sum of electronic and thermal Enthalpies=	-1562.409914
Sum of electronic and thermal Free Energies=	-1562.452304

$CHCl_2SO_2N$-singlet-III-TS3S -1.05493900 0.06503200 0.39993400

O	-1.98315900	0.72429200	-1.14169100
O	-0.86383500	-0.18501200	1.81606400
C	0.58855000	0.04683300	-0.48546200
N	-2.07172800	-0.61186800	-0.55571100
Cl	1.57122800	1.40777200	0.03455500
Cl	1.38979300	-1.49752600	-0.23880300
H	0.28841000	0.17314700	-1.51895700

Zero-point correction=	0.028984
Thermal correction to Energy=	0.036474
Thermal correction to Enthalpy=	0.037418
Thermal correction to Gibbs Free Energy=	-0.004806
Sum of electronic and zero-point Energies=	-1562.406535
Sum of electronic and thermal Energies=	-1562.399046
Sum of electronic and thermal Enthalpies=	-1562.398101
Sum of electronic and thermal Free Energies=	-1562.440326

$CHCl_2SO_2N$-triplet-I

S	-1.11200700	-0.07556000	0.05452900
O	-1.77172600	-1.08314000	-0.72611100
O	-1.16669100	-0.06729300	1.48489100
C	0.64170600	-0.00644700	-0.52142200
H	0.61563600	-0.01878300	-1.60528000
Cl	1.45782100	-1.45922700	0.04868200
Cl	1.40065900	1.49206800	0.02340000
N	-1.68008300	1.41594100	-0.49061100

Zero-point correction=	0.030749
Thermal correction to Energy=	0.038388
Thermal correction to Enthalpy=	0.039332
Thermal correction to Gibbs Free Energy=	-0.004009
Sum of electronic and zero-point Energies=	-1562.478167
Sum of electronic and thermal Energies=	-1562.470528
Sum of electronic and thermal Enthalpies=	-1562.469584
Sum of electronic and thermal Free Energies=	-1562.512925

$CHCl_2SO_2N$-triplet-II

S	1.14339800	-0.00005000	-0.04856200
O	1.71190000	-1.25066300	-0.45877700
O	1.71204700	1.25037400	-0.45902100
C	-0.64138800	-0.00006600	-0.52392400
Cl	-1.43428100	1.47242000	0.04054900
N	1.07975500	0.00013500	1.64208100
H	-0.62639000	0.00000500	-1.61066400
Cl	-1.43451300	-1.47226900	0.04057000

Zero-point correction=	0.030578
Thermal correction to Energy=	0.038239
Thermal correction to Enthalpy=	0.039183

Thermal correction to Gibbs Free Energy= -0.004408
Sum of electronic and zero-point Energies= -1562.477171
Sum of electronic and thermal Energies= -1562.469510
Sum of electronic and thermal Enthalpies= -1562.468566
Sum of electronic and thermal Free Energies= -1562.512157

$CHCl_2NSO_2$-I

S	-1.69338900	-0.00007000	-0.01874600
O	-1.92409800	0.00050100	1.39600300
O	-2.77532800	-0.00029300	-0.94522300
C	0.83641800	-0.00024600	0.25914000
H	0.60108200	0.00010400	1.31650800
N	-0.30521400	-0.00041000	-0.61224300
Cl	1.80005100	1.47217700	-0.05582800
Cl	1.80033300	-1.47195900	-0.05546300

Zero-point correction= 0.033459
Thermal correction to Energy= 0.040732
Thermal correction to Enthalpy= 0.041676
Thermal correction to Gibbs Free Energy= -0.000745
Sum of electronic and zero-point Energies= -1562.568738
Sum of electronic and thermal Energies= -1562.561465
Sum of electronic and thermal Enthalpies= -1562.560521
Sum of electronic and thermal Free Energies= -1562.602942

$CHCl_2NSO_2$-I-TS4

S	1.88937200	-0.06459400	0.00446200
O	1.36589800	0.72923900	-1.08843800
O	3.31412200	-0.26880200	0.02775300
C	-1.42819900	-0.11809500	0.57647600
N	1.02094600	-0.65283900	1.05509100
Cl	-1.71487200	1.47225500	0.15937100
Cl	-2.15974200	-1.29474300	-0.36735400
H	-0.37911200	-0.38925300	1.10530000

Zero-point correction= 0.025753
Thermal correction to Energy= 0.033860
Thermal correction to Enthalpy= 0.034804
Thermal correction to Gibbs Free Energy= -0.011251
Sum of electronic and zero-point Energies= -1562.469816
Sum of electronic and thermal Energies= -1562.461709
Sum of electronic and thermal Enthalpies= -1562.460765

Sum of electronic and thermal Free Energies= -1562.506820

$CHCl_2NSO_2$-II

S	1.49470300	0.00001600	0.02698600
O	1.22379300	-0.00001000	1.42938800
O	2.82894800	0.00001300	-0.47376900
C	-0.98452500	0.00000200	-0.71878600
N	0.41088800	0.00001800	-1.02543400
Cl	-1.52329300	1.47985300	0.14927200
Cl	-1.52324400	-1.47987700	0.14925700
H	-1.51510500	-0.00000200	-1.66096700

Zero-point correction= 0.033451
Thermal correction to Energy= 0.040699
Thermal correction to Enthalpy= 0.041644
Thermal correction to Gibbs Free Energy= -0.000203
Sum of electronic and zero-point Energies= -1562.567606
Sum of electronic and thermal Energies= -1562.560358
Sum of electronic and thermal Enthalpies= -1562.559414
Sum of electronic and thermal Free Energies= -1562.601260

$CHCl_2S(O)NO$-syn-I

S	0.69771500	-0.46955600	0.75875800
O	1.31722000	-1.66198600	0.14186300
C	-0.67731900	0.09448300	-0.36289900
H	-0.32391900	0.14841000	-1.38689700
N	2.04232700	0.96759400	-0.20350200
Cl	-1.15455600	1.71511500	0.18031700
Cl	-2.01628300	-1.06734500	-0.32079900
O	2.78682600	0.28852900	-0.73725400

Zero-point correction= 0.029885
Thermal correction to Energy= 0.038363
Thermal correction to Enthalpy= 0.039307
Thermal correction to Gibbs Free Energy= -0.005259
Sum of electronic and zero-point Energies= -1562.525252
Sum of electronic and thermal Energies= -1562.516774
Sum of electronic and thermal Enthalpies= -1562.515830
Sum of electronic and thermal Free Energies= -1562.560396

$CHCl_2S(O)NO$-syn-II

S	-0.55433200	-1.11400800	-0.14798200
O	-0.96093000	-1.03457100	1.26787900
C	0.75852300	0.16343300	-0.50953000
N	-2.08901700	0.22401500	-0.92399100
Cl	2.36272500	-0.50843300	-0.11840000
O	-2.77274500	0.38448900	-0.02544300
Cl	0.46569200	1.69309200	0.32631500
H	0.72760100	0.33688400	-1.58121200

Zero-point correction= 0.029777
Thermal correction to Energy= 0.038296
Thermal correction to Enthalpy= 0.039240
Thermal correction to Gibbs Free Energy= -0.005562
Sum of electronic and zero-point Energies= -1562.522939
Sum of electronic and thermal Energies= -1562.514419
Sum of electronic and thermal Enthalpies= -1562.513475
Sum of electronic and thermal Free Energies= -1562.558278

$CHCl_2S(O)NO$-syn-III

S	-0.71839800	-0.94365200	0.58840600
O	-1.75357200	-0.15913500	1.29649000
C	0.87763200	0.02503600	0.59650400
N	-1.25111100	-0.22287000	-1.41695600
O	-2.20875600	0.35760900	-1.21852500
H	1.27985500	-0.00752300	1.60279100
Cl	1.98922800	-0.85520700	-0.47543200
Cl	0.68166200	1.73332700	0.16358800

Zero-point correction= 0.029936
Thermal correction to Energy= 0.038387
Thermal correction to Enthalpy= 0.039331
Thermal correction to Gibbs Free Energy= -0.004943
Sum of electronic and zero-point Energies= -1562.525405
Sum of electronic and thermal Energies= -1562.516955
Sum of electronic and thermal Enthalpies= -1562.516010
Sum of electronic and thermal Free Energies= -1562.560284

$CHCl_2S(O)NO$-anti-I

S	-0.65879000	-1.07950800	0.08443600
O	-0.52019800	-1.32632200	1.52490000
C	0.54607800	0.25401100	-0.42968900
N	-2.23472200	0.31502600	-0.12504400

Cl	2.15099200	-0.47446100	-0.66621300
O	-2.45328800	0.57704700	-1.22694900
Cl	0.58357400	1.58677100	0.73153600
H	0.20750000	0.62780500	-1.39163300

Zero-point correction= 0.029493
Thermal correction to Energy= 0.038154
Thermal correction to Enthalpy= 0.039098
Thermal correction to Gibbs Free Energy= -0.006136
Sum of electronic and zero-point Energies= -1562.516977
Sum of electronic and thermal Energies= -1562.508317
Sum of electronic and thermal Enthalpies= -1562.507373
Sum of electronic and thermal Free Energies= -1562.552606

$CHCl_2S(O)NO$-anti-II

S	-1.05013400	-0.15047900	-0.85238800
O	-1.77626900	-1.42141300	-0.67073000
C	0.75136200	-0.47785200	-0.48948000
N	-1.35566900	0.79097100	1.06296100
O	-1.24150200	1.92947600	1.08630100
H	1.10947100	-1.15754900	-1.25518200
Cl	1.61062400	1.06904100	-0.66603400
Cl	1.02563200	-1.25545100	1.08162100

Zero-point correction= 0.029439
Thermal correction to Energy= 0.038174
Thermal correction to Enthalpy= 0.039119
Thermal correction to Gibbs Free Energy= -0.006348
Sum of electronic and zero-point Energies= -1562.518534
Sum of electronic and thermal Energies= -1562.509799
Sum of electronic and thermal Enthalpies= -1562.508855
Sum of electronic and thermal Free Energies= -1562.554322

$CHCl_2S(O)NO$-anti-III

S	-0.59365500	-0.79985300	-0.54051600
O	-0.69534200	-2.11923900	0.11871200
C	0.69200700	0.16450800	0.37996400
N	-2.04917700	0.24514700	0.61927000
O	-2.99053600	0.57061700	0.05251900
Cl	0.56877000	1.84875600	-0.17721900
H	0.46162600	0.12568500	1.43998300
Cl	2.29688100	-0.53358700	0.13155800

Zero-point correction=	0.029466
Thermal correction to Energy=	0.038213
Thermal correction to Enthalpy=	0.039157
Thermal correction to Gibbs Free Energy=	-0.006387
Sum of electronic and zero-point Energies=	-1562.520298
Sum of electronic and thermal Energies=	-1562.511551
Sum of electronic and thermal Enthalpies=	-1562.510607
Sum of electronic and thermal Free Energies=	-1562.556151

CHClNCl-anti

C	0.00000000	0.56575300	0.00000000
H	-0.91841500	1.14223100	0.00000000
Cl	1.43545500	1.52046300	0.00000000
N	0.10805900	-0.68956500	0.00000000
Cl	-1.42592600	-1.50339200	0.00000000

Zero-point correction=	0.021554
Thermal correction to Energy=	0.025956
Thermal correction to Enthalpy=	0.026900
Thermal correction to Gibbs Free Energy=	-0.006356
Sum of electronic and zero-point Energies=	-1013.876296
Sum of electronic and thermal Energies=	-1013.871893
Sum of electronic and thermal Enthalpies=	-1013.870949
Sum of electronic and thermal Free Energies=	-1013.904205

CHClNCl-syn

C	0.00000000	1.11815700	0.00000000
N	-1.11624400	0.53394800	0.00000000
Cl	-1.13064100	-1.18334300	0.00000000
Cl	1.59064700	0.43935900	0.00000000
H	-0.00638700	2.20116000	0.00000000

Zero-point correction=	0.022010
Thermal correction to Energy=	0.026144
Thermal correction to Enthalpy=	0.027088
Thermal correction to Gibbs Free Energy=	-0.006095
Sum of electronic and zero-point Energies=	-1013.878321
Sum of electronic and thermal Energies=	-1013.874187
Sum of electronic and thermal Enthalpies=	-1013.873243
Sum of electronic and thermal Free Energies=	-1013.906427

$HNSO_2$

S	-0.02798700	-0.00012900	-0.00051800
O	-0.35617900	1.39587300	0.00028200
O	-1.05954200	-0.98578800	0.00047200
H	2.04229400	0.27506700	0.00219600
N	1.39018000	-0.50766900	0.00000900

Zero-point correction=	0.023646
Thermal correction to Energy=	0.027412
Thermal correction to Enthalpy=	0.028356
Thermal correction to Gibbs Free Energy=	-0.002868
Sum of electronic and zero-point Energies=	-604.016911
Sum of electronic and thermal Energies=	-604.013144
Sum of electronic and thermal Enthalpies=	-604.012200
Sum of electronic and thermal Free Energies=	-604.043425

O_2SN

S	0.00000000	0.01331300	-0.00016100
O	-1.24885200	-0.68306300	0.00011000
O	1.24885300	-0.68306200	0.00011000
N	-0.00000100	1.53085700	0.00011500

Zero-point correction=	0.011125
Thermal correction to Energy=	0.014882
Thermal correction to Enthalpy=	0.015827
Thermal correction to Gibbs Free Energy=	-0.016028
Sum of electronic and zero-point Energies=	-603.354202
Sum of electronic and thermal Energies=	-603.350444
Sum of electronic and thermal Enthalpies=	-603.349500
Sum of electronic and thermal Free Energies=	-603.381355

$OCCl_2$

C	0.00000000	0.00000000	0.49907100
O	0.00000000	0.00000000	1.67207500
Cl	0.00000000	-1.45288900	-0.48150100
Cl	0.00000000	1.45288900	-0.48150100

Zero-point correction=	0.010433
Thermal correction to Energy=	0.014380
Thermal correction to Enthalpy=	0.015324
Thermal correction to Gibbs Free Energy=	-0.016848

Sum of electronic and zero-point Energies=	-1033.819066
Sum of electronic and thermal Energies=	-1033.815119
Sum of electronic and thermal Enthalpies=	-1033.814174
Sum of electronic and thermal Free Energies=	-1033.846347

HNSO

N	-1.29072900	0.38642000	0.00000000
S	0.00000000	-0.40037800	0.00000000
O	1.28216600	0.28694100	0.00000000
H	-1.22222600	1.40557100	0.00000000

Zero-point correction=	0.018310
Thermal correction to Energy=	0.021571
Thermal correction to Enthalpy=	0.022515
Thermal correction to Gibbs Free Energy=	-0.006697
Sum of electronic and zero-point Energies=	-528.800868
Sum of electronic and thermal Energies=	-528.797607
Sum of electronic and thermal Enthalpies=	-528.796663
Sum of electronic and thermal Free Energies=	-528.825875

CH_2Cl

C	0.00000000	0.00000000	-1.11368200
H	0.00000000	-0.95126300	-1.61552300
H	0.00000000	0.95126300	-1.61552300
Cl	0.00000000	0.00000000	0.58312600

Zero-point correction=	0.022737
Thermal correction to Energy=	0.026200
Thermal correction to Enthalpy=	0.027144
Thermal correction to Gibbs Free Energy=	-0.000839
Sum of electronic and zero-point Energies=	-499.468085
Sum of electronic and thermal Energies=	-499.464622
Sum of electronic and thermal Enthalpies=	-499.463678
Sum of electronic and thermal Free Energies=	-499.491661

CCl_2H

C	0.00000000	0.00000000	0.66754900
H	0.00000000	0.00000000	1.74258700
Cl	0.00000000	1.47388200	-0.16905500
Cl	0.00000000	-1.47388200	-0.16905500

Zero-point correction=	0.014707
Thermal correction to Energy=	0.018109
Thermal correction to Enthalpy=	0.019053
Thermal correction to Gibbs Free Energy=	-0.011790
Sum of electronic and zero-point Energies=	-959.104322
Sum of electronic and thermal Energies=	-959.100920
Sum of electronic and thermal Enthalpies=	-959.099976
Sum of electronic and thermal Free Energies=	-959.130818

HCN

H	0.00000000	0.00000000	-1.56260200
C	0.00000000	0.00000000	-0.49689400
N	0.00000000	0.00000000	0.64913800

Zero-point correction=	0.016299
Thermal correction to Energy=	0.018847
Thermal correction to Enthalpy=	0.019791
Thermal correction to Gibbs Free Energy=	-0.003061
Sum of electronic and zero-point Energies=	-93.444370
Sum of electronic and thermal Energies=	-93.441822
Sum of electronic and thermal Enthalpies=	-93.440878
Sum of electronic and thermal Free Energies=	-93.463730

ClCN

C	0.00000000	0.00000000	-0.65491400
N	0.00000000	0.00000000	-1.80766500
Cl	0.00000000	0.00000000	0.97547900

Zero-point correction=	0.008834
Thermal correction to Energy=	0.011888
Thermal correction to Enthalpy=	0.012832
Thermal correction to Gibbs Free Energy=	-0.013838
Sum of electronic and zero-point Energies=	-553.070479
Sum of electronic and thermal Energies=	-553.067424
Sum of electronic and thermal Enthalpies=	-553.066480
Sum of electronic and thermal Free Energies=	-553.093150

CHCl

C	0.04504100	1.19068000	0.00000000
H	-1.03594200	1.43051100	0.00000000
Cl	0.04504100	-0.50438800	0.00000000

Zero-point correction=	0.011301
Thermal correction to Energy=	0.014226
Thermal correction to Enthalpy=	0.015170
Thermal correction to Gibbs Free Energy=	-0.011476
Sum of electronic and zero-point Energies=	-498.803291
Sum of electronic and thermal Energies=	-498.800366
Sum of electronic and thermal Enthalpies=	-498.799421
Sum of electronic and thermal Free Energies=	-498.826067

CCl_2

C	0.00000000	0.00000000	0.84096800
Cl	0.00000000	-1.41017400	-0.14840600
Cl	0.00000000	1.41017400	-0.14840600

Zero-point correction=	0.004056
Thermal correction to Energy=	0.007475
Thermal correction to Enthalpy=	0.008419
Thermal correction to Gibbs Free Energy=	-0.021661
Sum of electronic and zero-point Energies=	-958.464542
Sum of electronic and thermal Energies=	-958.461124
Sum of electronic and thermal Enthalpies=	-958.460179
Sum of electronic and thermal Free Energies=	-958.490259
